# Stepwise Operation of a Molecular Rotary Motor Driven by an Appel Reaction

**DOI:** 10.1021/jacs.3c10266

**Published:** 2024-02-06

**Authors:** Patrick Zwick, Axel Troncossi, Stefan Borsley, Iñigo J. Vitorica-Yrezabal, David A. Leigh

**Affiliations:** † Department of Chemistry, University of Manchester, Oxford Road, Manchester M13 9PL, United Kingdom; ‡ School of Chemistry and Molecular Engineering, East China Normal University, Shanghai 200062, China

## Abstract

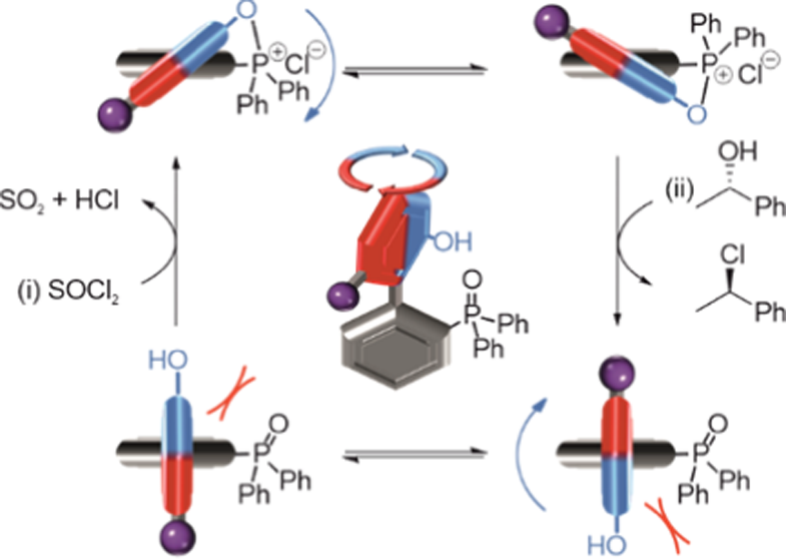

To date, only a small number of chemistries and chemical fueling strategies have been successfully used to operate artificial molecular motors. Here, we report the 360° directionally biased rotation of phenyl groups about a C–C bond, driven by a stepwise Appel reaction sequence. The motor molecule consists of a biaryl-embedded phosphine oxide and phenol, in which full rotation around the biaryl bond is blocked by the P–O oxygen atom on the rotor being too bulky to pass the oxygen atom on the stator. Treatment with SOCl_2_ forms a cyclic oxyphosphonium salt (removing the oxygen atom of the phosphine oxide), temporarily linking the rotor with the stator. Conformational exchange via ring flipping then allows the rotor and stator to twist back and forth past the previous limit of rotation. Subsequently, the ring opening of the tethered intermediate with a chiral alcohol occurs preferentially through a nucleophilic attack on one face. Thus, the original phosphine oxide is reformed with net directional rotation about the biaryl bond over the course of the two-step reaction sequence. Each repetition of SOCl_2_–chiral alcohol additions generates another directionally biased rotation. Using the same reaction sequence on a derivative of the motor molecule that forms atropisomers rather than fully rotating 360° results in enantioenrichment, suggesting that, on average, the motor molecule rotates in the “wrong” direction once every three fueling cycles. The interconversion of phosphine oxides and cyclic oxyphosphonium groups to form temporary tethers that enable a rotational barrier to be overcome directionally adds to the strategies available for generating chemically fueled kinetic asymmetry in molecular systems.

## Introduction

Artificial molecular motors have been developed^1^ that transduce energy^2^ from various sources (including chemical reagents,^2c,3^ light,^4^ and electricity^5^) into directionally biased dynamics. The earliest synthetic chemically driven motor molecules required up to 8-step reaction sequences for their operation.^3b,3c^ Simpler “pulsed fuel” systems^3i,6^ (one fuel pulse required for each cycle of the motor) were subsequently developed, and the first autonomous artificial chemically fueled motor molecules based, like biomolecular motors,^7^ on the motor’s catalysis^3e,3g,3k−3o,8^ of fuel-to-waste reactions^2c^ have recently been realized.^2b^ However, the design of effective approaches for the chemical fueling of artificial molecular motors remains challenging, and only a limited number of chemistries have been successively developed to date for this purpose.^2c^

Biasing the direction of rotation around a covalent single bond provides a demanding test for both operational strategies and chemistries for rotary motors.^3a,3c,3d,3h,3j,3m,9^ The temporary formation of a tether between the rotor and stator to modulate a rotational barrier depending on the chemical state (i.e., mechanical gating;^2c^ having different conformations accessible at different stages of a sequence of chemical transformations) was first explored in a triptycene-based helicene by Kelly.^3a^ A reaction sequence involving (i) formation of the tether, (ii) directional rotation over an energy barrier to a new energy minimum, and (iii) cleavage of the tether to regenerate the original functional groups successfully led to the directional 120° rotation of the triptycene rotor about the helicene stator. However, Kelly was never able to extend this strategy to 360° rotation, nor to carry out the required chemical transformations autonomously (i.e., directional rotation of the motor continuing until the fuel supply is exhausted).^3d^

A consideration of the performance and mode of operation of various, recently designed ratchets suggests that transformations during the fuel-to-waste conversion in the chemomechanical cycle^2^ (a reaction cycle that involves different constitutional and conformational states) of a chemically fueled rotary motor should satisfy a number of criteria:^2c,10^

(i) The fuel-to-waste conversion must be overall exergonic, as directionally biasing stochastic processes can, in principle, be used to perform work (e.g., to wind up and apply strain to a polymer chain).^1a^
(ii) The reaction sequence must form an intermediate of the motor molecule (motor′) and subsequently regenerate the original form of the motor by a nonreciprocal pathway. This constitutes a chemical engine cycle (a chemomechanical cycle in which a catalyst transduces energy from a fuel-to-waste reaction it promotes).^2b^
(iii) Unreactive byproducts of the motor molecule should be minimalized so that the motor is not depleted within a few reaction cycles.^11^
(iv) The two chemical states, motor and motor′, must access a different range of conformational dynamics (mechanical gating^2c^) and the different conformations must react within the engine cycle at different rates (chemical gating,^2e,8^ effectively the Curtin–Hammett principle^2e^).

**Figure 1:**
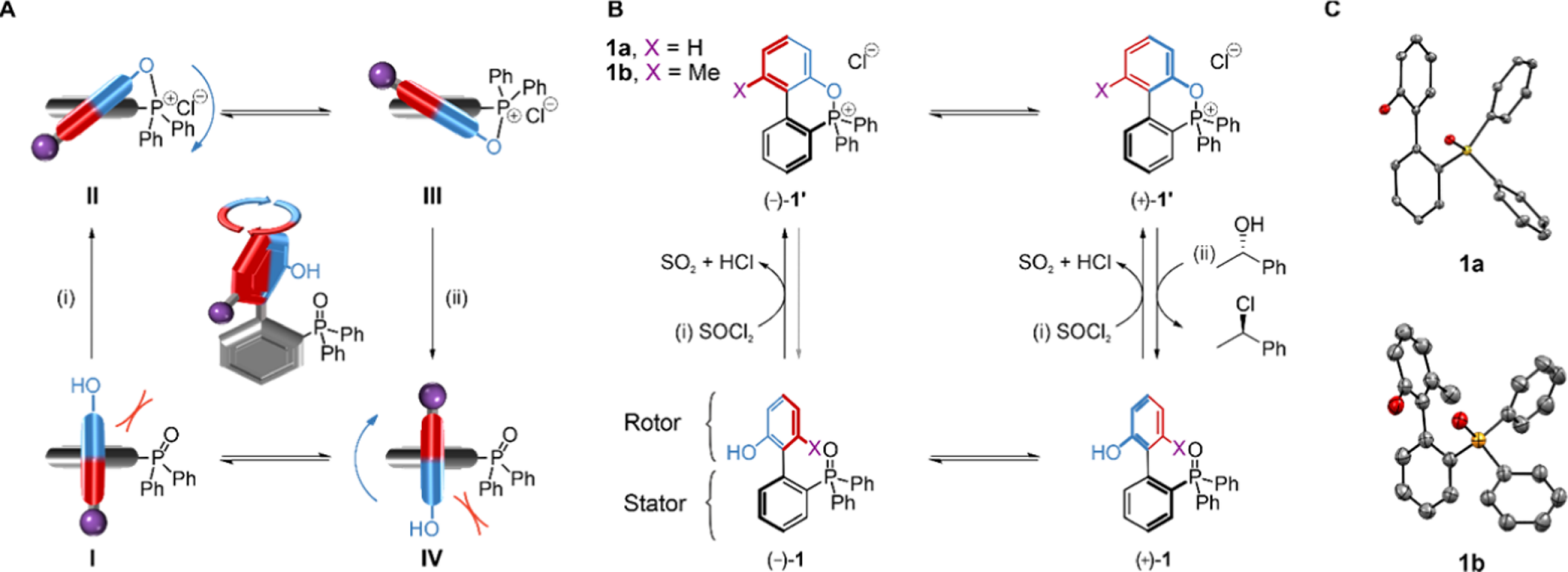
(A) Schematic representation of chemically powered rotation of the rotor about the stator in a phosphine oxide–phenol-based rotary molecular motor. (B) Chemomechanical cycle for **1**. Reaction of (±)-**1** with SOCl_2_ forms cyclic phosphonium (±)-**1′**, which undergoes dynamic interconversion by a ring flip. Ring opening with a chiral alcohol (e.g., (*S*)-1-phenylethan-1-ol) preferentially reforms (+)-**1** through dynamic kinetic resolution. Under fueling, **1a** (unsubstituted at the 6-position of the rotor, X = H) undergoes directionally biased 360° rotation, while atropisomers (+)-**1b** and (−)-**1b** (methylated at the 6-position of the rotor, X = Me) become enantioenriched. (C) Oak ridge thermal-ellipsoid plot (ORTEP) of the X-ray crystal structures of **1a** and **1b**. Thermal ellipsoids are shown at the 50% probability level. Carbon atoms are colored gray, oxygen atoms are colored red, and phosphorus atoms are colored yellow. Hydrogen atoms are omitted for clarity.

Phosphine oxides have been used as mediators in a number of challenging nucleophilic displacement processes,^12^ including Appel^12a−12e^ and Mitsunobu reactions.^12f^ Denton’s catalytic Mitsunobu protocol^12f^ involves the transformation of a phosphine oxide into a cyclic oxyphosphonium salt, which we rationalized might be adapted to serve as a tether within a rotary motor (Figure 1). While the elevated temperatures and Dean–Stark conditions routinely required for Mitsunobu reactions are poorly suited for driving a motor,^2c^ the same type of oxyphosphonium intermediates can be formed at lower temperatures in Appel reactions.^12a−12e^

## Experimental Design

To explore this chemistry for motor molecule applications, we prepared biaryl compounds **1a** and **1b**,^13^ which bear a phosphine oxide on the 2-position of the stator and a OH group on the 2-position of the rotor (Figure 1B). The 6-position of the rotor is either unsubstituted (**1a**, X = H) or substituted with a methyl group (**1b**). The solid-state structures of both compounds were determined by single-crystal X-ray crystallography (Figure 1C and Supporting Information, Section S6). From the X-ray structures, **1a** and **1b** are structurally very similar to each other, making **1b** a suitable model for evaluating the operation of motor molecule **1a**.

Molecular models suggested that, for steric reasons, neither the rotor –OH group nor the methyl group should be able to pass the phosphine oxide substituent on the stator of **1b**, forming atropisomers. In contrast, it appeared that **1a** should not form kinetically stable atropisomers because the hydrogen in the 6-position of the rotor can freely pass the phosphine oxide substituent on the stator. However, the OH group in the 2-position of the rotor still cannot pass the phosphine oxide substituent of the stator.

We reasoned that the treatment of **1a/1b** with SOCl_2_ would form a 6-membered cyclic oxyphosphonium tether (**I/IV** → **II**/**III**), which might then allow passage of the *O*-substituent of the rotor past the stator through a ring flip.^3m^ Face-selective nucleophilic displacement of the oxyphosphonium group with a chiral alcohol would then regenerate the functional groups of the original motor (concomitant with the conversion of the alcohol to the corresponding alkyl chloride). This would lead to net-directional 360° rotation about the biaryl bond in the case of **1a** and enantioenrichment in the case of **1b** (i.e., **III** → **IV** is faster than **II** → **I**). In this way, directional rotation about the biaryl C–C bond of **1a** would be driven by a stepwise sequence of chemical and conformational changes (**I** → **II** → **III** → **IV** → **I**, Figure 1A).

To demonstrate the directional rotation of the motor molecules, we sought to experimentally verify (i) that the chemical transformations proceed as intended (i.e., formation and opening of the tether; **I/IV** → **II/III** and **II/III** → **I/IV**); (ii) the conformational restrictions and dynamics in the different chemical states of the motor (i.e., **1** (**I** ⇌ **IV**) and **1′** (**II** ⇌ **III**)); and (iii) the kinetic asymmetry (the tendency of a chemomechanical cycle to progress in one direction over the other when driven by a fuel-to-waste reaction as a consequence of kinetic gating) of the overall chemical engine cycle^2,8^ (determined by the difference in the rates of opening each atropisomeric conformation (**III** → **IV** versus **II** → **I**)).

## Results and Discussion

**Figure 2:**
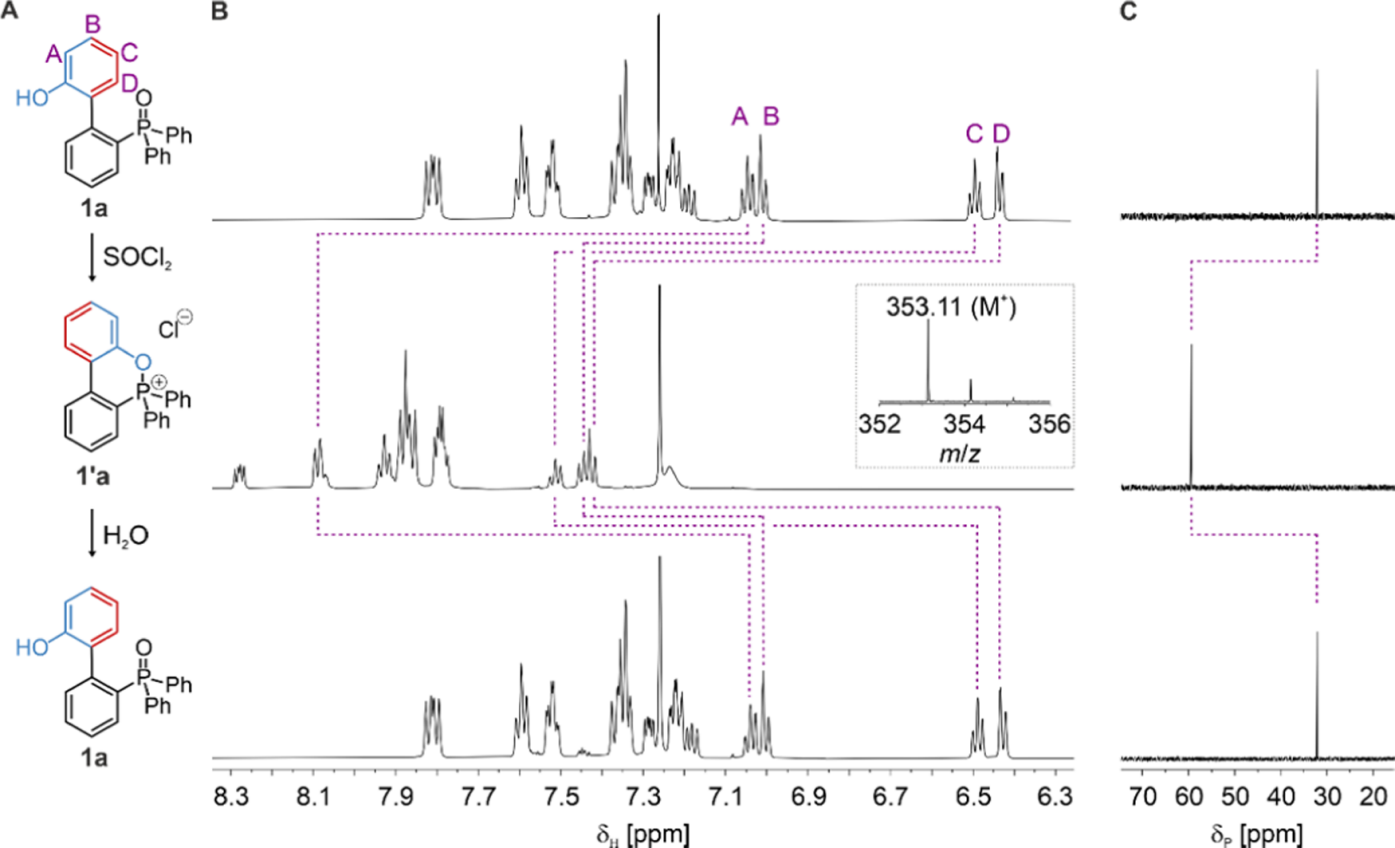
Chemical transformation of **1a** → **1′a** → **1a** upon treatment with SOCl_2_ followed by H_2_O in CDCl_3_ at room temperature, showing the formation and hydrolysis of the oxyphosphonium salt **1′a**. (A) Reaction scheme for the chemical transitions. (B) Partial ^1^H NMR (600 MHz, CDCl_3_, 293 K) spectra of **1a** (top), 1 h following the addition of SOCl_2_ to form **1′a** (middle), and 5 min following the subsequent addition of H_2_O to reform **1a** (bottom). Changes in the chemical shifts of protons A, B, C, and D are indicated by dashed lines. Inset shows the MALDI-ToF-MS spectrum of **1’a**. (C) Partial ^31^P{^1^H} NMR (126 MHz, CDCl_3_, 293 K) spectra of **1a** (top), 1 h following the addition of SOCl_2_ to form **1′a** (middle), and 5 min following the subsequent addition of H_2_O to reform **1a** (bottom).

We first investigated the chemical transformation of the phosphine oxide **1a** to the oxyphosphonium salt **1′a** and its subsequent hydrolysis (Figure 2).^12^ Pleasingly, the treatment of a CDCl_3_ solution of **1a** with SOCl_2_ ([**1a**] = 5 mM, [SOCl_2_] = 50 mM (10 equiv); Supporting Information, Section S4.1) quantitatively transformed **1a** to a single new species within 1 h. The ^31^P{^1^H} NMR spectrum (Figure 2C) of the product showed a single phosphorus resonance with a chemical shift of 60 ppm, typical of cyclic oxyphosphonium salts.^12f^ The identity of the phosphonium salt as **1′a** was confirmed by matrix-assisted laser-desorption ionization time-of-flight mass spectrometry (MALDI-ToF-MS; Figure 2B). The addition of H_2_O (50 mM) to the reaction mixture rapidly regenerated **1a** with less than 2% of other species (likely arising from side reactions of the motor during the reaction cycle). Similar results were obtained when **1a** was replaced by **1b** (Supporting Information, Section S4.2).

We next investigated the mechanical (i.e., conformational) transitions and dynamics in the putative motor molecules. As anticipated, **1b** forms atropisomers, which proved to be readily separable by chiral stationary phase high-performance liquid chromatography (chiral HPLC; Supporting Information, Section S3). In contrast, the lack of kinetically stable atropisomers for **1a** confirmed that the –OH of the rotor freely passes the hydrogen atom at the 6-position of the stator in **1a**. (Note that the observation that **1b** does form atropisomers indicates that the –OH of the rotor cannot pass the phosphine oxide at the 2-position in these systems, with a barrier at ∼136 kJ/mol, Supporting Information, Section S4.7.)

To explore whether the *O*-substituent of the rotor could pass the 2-position of the stator in the oxyphosphonium chemical state of the motor, enantiopure (−)-**1b** and (+)-**1b** were obtained by preparative chiral HPLC. Treatment of these samples with SOCl_2_, followed by H_2_O ((i) [(−)-**1b**/(+)-**1b**] = 5 mM, [SOCl_2_] = 50 mM (10 equiv) and (ii) H_2_O (50 mM); Supporting Information, Section S4.3), afforded racemic (±)-**1b**, confirming rapid dynamic exchange between (−)-**1′b** and (+)-**1′b** (Supporting Information, Section S4.3).

**Figure 3:**
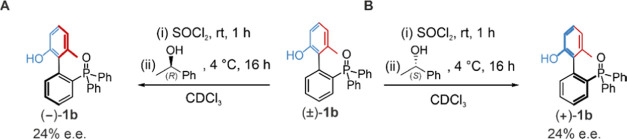
Directional operation of motor molecule **1b**. Treatment of racemic **1b** with SOCl_2_ followed by (*R*)-1-phenylethan-1-ol (**A**) or (*S*)-1-phenylethan-1-ol (**B**) results in the formation of enantioenriched (±)-**1b** in 24% e.e., as determined by chiral HPLC (see Supporting Information, Section S4.4 and S4.5).

Having demonstrated the necessary chemical and mechanical transitions, we next sought to introduce kinetic asymmetry^2,8^ into the chemical engine cycle through a dynamic kinetic resolution of the cyclic oxyphosphonium salt. Screening of a selection of commercially available chiral alcohols under various reaction conditions (varying solvent and temperature; Table S1, Supporting Information, Section S4.4) led to the following operating procedure: Treatment of a CDCl_3_ solution of racemic (±)-**1b** with SOCl_2_ ([(±)-**1b**] = 5 mM, [SOCl_2_] = 50 mM (10 equiv), 1 h) cleanly generated (±)-**1′b**. Cooling of the reaction mixture to 4 °C, followed by the addition of (*R*)-1-phenylethan-1-ol (10 equiv, 50 mM), resulted in an Appel reaction,^12a−12c^ forming (1-chloroethyl)benzene^14^ and regenerating (−)-**1b** in 24% e.e. after 16 h (Figure 3A; Supporting Information, Sections S4.4 and S4.5). Carrying out a similar reaction with (*S*)-1-phenylethan-1-ol instead of (*R*)-1-phenylethan-1-ol gave an equal enrichment of the opposite enantiomer, (+)-**1b** (Figure 3B). Molecular modeling indicates that the methyl group in **1′b** has a negligible effect on the conformation of the P–O ring (Supporting Information, Section S5), indicating that **1′b** is a suitable model for the stereochemical outcome of the Appel reaction promoted by **1′a**.

The reaction sequence was repeated 4× on **1a** without noticeable degradation of the motor molecule (Supporting Information, Section S4.6). Each repetition of the SOCl_2_–chiral alcohol reaction sequence (Figure 1B) generates an additional directionally biased rotation of the components. During the motor reaction cycle, the rotational dynamics are only gated (directionally biased) at the ring-opening step (i.e., the motor is singly kinetically gated); there is no directional bias in the closing of the tether, i.e., there is an equal probability of ring closing occurring from **I** → **II** or **IV** → **III**, Figure 1A. The 24% e.e. of each reaction cycle suggests that the first-generation phosphine oxide motor makes a “mistake” in the direction of full rotation roughly once every three rotations.

## Conclusions

In conclusion, 360° directional rotation about a covalent single bond can be driven through an information ratchet mechanism^1a,2,8^ by a suitable biaryl motor molecule’s promotion of an Appel reaction.^12a−12e^ Directionality (the overall ratio of forward-to-backward steps taken by the machine) of the singly kinetically gated motor arises from the enantioselective opening of the cyclic oxyphosphonium salt with a chiral alcohol. This expands the toolbox of strategies and chemistries available to drive directional molecular motion.^15^ However, the current system is restricted to stepwise operation because, when the motor, chiral alcohol, and SOCl_2_ are present contemporaneously, the alcohol preferentially reacts with SOCl_2_ rather than with the motor. Work is ongoing to develop the use of catalytic analogs^12a−12e^ of Appel-like reactions with phosphine oxide-based motors, as required for autonomous chemical fueling.^3g,3k−3o^

## Supporting Information Available

Experimental methods and operations data and X-ray crystal structure determination method and data is provided (PDF)

## Abbreviations

HPLC: high-performance liquid chromatography
MALDI-ToF-MS: matrix-assisted laser-desorption ionization time-of-flight mass spectrometry
NMR: nuclear magnetic resonance

## References

[ref1] aKay E. R.; Leigh D. A.; Zerbetto F. Synthetic molecular motors and mechanical machines. Angew. Chem., Int. Ed. 2007, 46, 72–191. 10.1002/anie.200504313. 17133632

[ref2] aRagazzon G.; Prins L. J. Energy consumption in chemical fuel-driven self-assembly. Nat. Nanotechnol. 2018, 13, 882–889. 10.1038/s41565-018-0250-8. 30224796

[ref3] aKelly T. R.; De Silva H.; Silva R. A. Unidirectional rotary motion in a molecular system. Nature 1999, 401, 150–152. 10.1038/43639. 10490021

[ref4] aKoumura N.; Zijlstra R. W. J.; van Delden R. A.; Harada N.; Feringa B. L. Light-driven monodirectional molecular rotor. Nature 1999, 401, 152–155. 10.1038/43646. 10490022

[ref5] aPumm A.-K.; Engelen W.; Kopperger E.; Isensee J.; Vogt M.; Kozina V.; Kube M.; Honemann M. N.; Bertosin E.; Langecker M.; Golestanian R.; Simmel F. C.; Dietz H. A DNA origami rotary ratchet motor. Nature 2022, 607, 492–498. 10.1038/s41586-022-04910-y. 35859200 PMC9300469

[ref6] aBiagini C.; Di Stefano S. Abiotic chemical fuels for the operation of molecular machines. Angew. Chem., Int. Ed. 2020, 59, 8344–8354. 10.1002/anie.201912659. 31898850

[ref7] Schliwa M.; Woehlke G. Molecular motors. Nature 2003, 422, 759–765. 10.1038/nature01601. 12700770

[ref8] Astumian R. D. Kinetic asymmetry allows macromolecular catalysts to drive an information ratchet. Nat. Commun. 2019, 10, 3837 10.1038/s41467-019-11402-7. 31444340 PMC6707331

[ref9] aDahl B. J.; Branchaud B. P. Synthesis and characterization of a functionalized chiral biaryl capable of exhibiting unidirectional bond rotation. Tetrahedron Lett. 2004, 45, 9599–9602. 10.1016/j.tetlet.2004.10.147.

[ref10] aSingh N.; Formon G. J. M.; De Piccoli S.; Hermans T. M. Devising synthetic reaction cycles for dissipative nonequilibrium self-assembly. Adv. Mater. 2020, 32, 1906834 10.1002/adma.201906834. 32064688

[ref11] Chen X.; Soria-Carrera H.; Zozulia O.; Boekhoven J. Suppressing catalyst poisoning in the carbodiimide-fueled reaction cycle. Chem. Sci. 2023, 14, 12653–12660. 10.1039/D3SC04281B. 38020366 PMC10646924

[ref12] aDenton R. M.; Ana J.; Adenirana B. Phosphine oxide-catalysed chlorination reactions of alcohols under Appel conditions. Chem. Commun. 2010, 46, 3025–3027. 10.1039/c002825h. 20386856

[ref13] Zhang H.-Y.; Yi H.-M.; Wang G.-W.; Yang B.; Yang S.-D. Pd(II)-Catalyzed C(sp^2^)–H hydroxylation with R_2_(O)P-coordinating group. Org. Lett. 2013, 15, 6186–6189. 10.1021/ol403028a. 24206176

[ref14] Measurement of the e.e. of the (1-chloroethyl)benzene product of the Appel reaction is complicated by the nonmotor-molecule-mediated chlorination with SOCl_2_ proceeding with retention.

[ref15] aBorsley S.; Leigh D. A.; Roberts B. M. W.; Vitorica-Yrezabal I. J. Tuning the force, speed, and efficiency of an autonomous chemically fueled information ratchet. J. Am. Chem. Soc. 2022, 144, 17241–17248. 10.1021/jacs.2c07633. 36074864 PMC9501901

