# Supplementary material for: Stepwise Operation of a Molecular Rotary Motor Driven by an Appel Reaction
Source: J Am Chem Soc. 2024 Feb 6;146(7):4467–72. doi: 10.1021/jacs.3c10266 (PMC10885133; doi:10.1021/jacs.3c10266)
Supplement: Supplementary file 1 — ja3c10266_si_001.pdf [file ja3c10266_si_001.pdf]

Stepwise operation of a molecular rotary motor driven by the Appel reaction

Patrick Zwick,[1] Axel Troncossi,[1] Stefan Borsley,[1] Iñigo J. Vitorica-Yrezabal[1] and David A. Leigh*[1,2]

[1]Department of Chemistry, University of Manchester, Oxford Road, Manchester, M13 9PL, United Kingdom.

[2]School of Chemistry and Molecular Engineering, East China Normal University, 200062 Shanghai, China.

*E-mail: david.leigh@manchester.ac.uk

– Supplementary Information –

Contents

S1. General methods and abbreviations....3

S2. Synthesis and characterization....4

S2.2 Synthesis and characterization of **1a**....4

S2.3 Synthesis and characterization of **1b**....5

S2.4 Synthesis and characterization of **1'a**....6

S2.5 Synthesis and characterization of **1'b**....7

S3. Chiral HPLC analysis of **1a** and **1b**....8

S4. Motor operations....9

S4.1 General procedure....9

S4.2 ^{1}H and ^{31}P{^{1}H} NMR spectra of closing **1b** followed by H_2O hydrolysis....10

S4.3 Chemically-driven racemisation of (+)- and (−)-**1b**....11

S4.4 Directional opening of **1'b**....12

S4.5 ^{1}H NMR analysis of directional opening of **1'b**....13

S4.6 ^{1}H NMR analysis of consecutively operating **1a**....14

S4.7 Thermal racemization of **1b**....15

S4.8 Proposed detailed mechanism of chemical transitions....16

S5. Molecular modelling of **1'a** and **1'b**....17

S6. Crystallographic data....18

S7. Spectroscopic data....21

S8. References....37

S1. General methods and abbreviations

Unless stated otherwise, reagents were obtained from commercial sources and used without purification. All chemicals, reagents, were purchased from Sigma Aldrich, UK (Merck KgaA) or Fluorochem UK. Deionized water was obtained by a milli-Q water purifier (Millipore). Anhydrous solvents were obtained by passing the solvent through an activated alumina column on a Phoenix SDS (solvent drying system; JC Meyer Solvent Systems, CA, USA). ^{1}H and ^{13}C{^{1}H}} NMR spectra were recorded on a Bruker Avance III instrument with an Oxford AS600 magnet equipped with a cryoprobe [5 mm CPDCH ^{13}C-^{1}H/D] (600 MHz) and ^{31}P{^{1}H} NMR spectra were recorded on a Bruker Avance III instrument with an B400 magnet (400 MHz) at a constant temperature of 20 °C. ^{1}H, ^{13}C, and ^{31}P chemical shifts are reported in parts per million (ppm) from low to high field and referenced to the literature values for chemical shifts of residual non-deuterated solvent, with respect to tetramethylsilane (0.00 ppm) as an external standard, for ^{1}H and ^{13}C NMRs. Standard abbreviations indicating multiplicity are used as follows: s (singlet), bs (broad singlet), d (doublet), t (triplet), dd (doublet of doublets), ddd (doublet of doublets of doublets), ddt (doublet of doublets of triplets), m (multiplet), *J* (coupling constant – quoted in Hz). All spectra were analyzed using MestReNova (Version 14.1.2). Fully characterized compounds were chromatographically homogeneous. Flash column chromatography was carried out using Silica 60 Å (particle size 40–63 µm, Sigma Aldrich, UK) as the stationary phase. TLC was performed on precoated silica gel plates (0.25 mm thick, 60 F254, Merck, Germany) and visualized using both short and long wave ultraviolet light in combination with standard laboratory stains (basic potassium permanganate, acidic ammonium molybdate and ninhydrin). Low resolution ESI mass spectrometry was performed with a Thermo Scientific LCQ Fleet Ion Trap Mass Spectrometer or an Agilent Technologies 1200 LC system with an Advion Expression LCMS single quadrupole MS detector. High-resolution mass spectrometry (HRMS) was carried out at the Mass Spectrometry Service, Department of Chemistry, University of Manchester. Matrix-assisted laser-desorption ionization (MALDI) mass spectrometry was performed with a Brucker rapiflex using dithernol as a matrix. Chiral high performance liquid chromatography (HPLC) was performed on an Agilent 1260 Infinity system. Column and conditions are specified below. HPLC data were analysed in Open Labs CDS software, and traces were exported as .csv data files for further plotting in OriginPro 2021b.

Abbreviations: COSY: correlated spectroscopy; DEPT: distortionless enhancement by polarization transfer; ESI: electrospray ionization; MALDI: matrix-assisted laser-desorption ionization; h: hour; HMBC: heteronuclear multiple bond correlation; HRMS: high-resolution mass spectrometry; HSQC: heteronuclear single quantum coherence; NMR: nuclear magnetic resonance; ppm: parts per million.

S2. Synthesis and characterization

S2.2 Synthesis and characterization of 1a

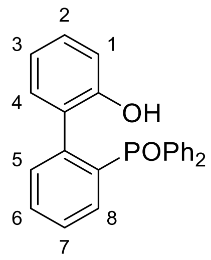

1a

Compound **1a** was synthesized according to previously reported procedures.[S1]

^{1}H NMR ($CDCl_3$, 600 MHz, 295 K): δ 8.99 (s, 1H, OH), 7.83 – 7.78 (m, 2H, $H_{7,8}$), 7.61 – 7.57 (m, 2H, H_{Ar}), 7.54 – 7.49 (m, 2H, $H_{5,6}$), 7.39 – 7.31 (m, 4H, H_{Ar}), 7.30 – 7.26 (m, 1H, H_{Ar}), 7.24 – 7.16 (m, 3H, H_{Ar}), 7.04 (ddd, *J* = 8.7, 7.1, 1.7 Hz, 1H, H_2), 7.00 (dd, *J* = 8.2, 1.4 Hz, 1H, H_1), 6.49 (td, J = 7.3, 1.4 Hz, 1H, H_3), 6.43 (dd, *J* = 7.6, 1.7 Hz, 1H, H_4).

^{31}P{^{1}H} NMR ($CDCl_3$, 400 MHz, 295 K): δ 32.17.

^{13}C{^{1}H} NMR ($CDCl_3$, 151 MHz, 295 K): δ 154.26, 144.53 – 144.47 (d, *J* = 8.6 Hz), 133.17 – 133.10 (d, *J* = 9.9 Hz), 132.95 – 132.92 (d, *J* = 3.9 Hz), 132.80 – 132.78 (d, *J* = 2.4 Hz), 132.70 – 132.62 (d, *J* = 12.7 Hz), 132.50 – 132.44 (d, *J* = 9.4 Hz), 132.39 – 132.38 (d, *J* = 2.8 Hz), 132.19 – 131.51 (d, *J* = 102.7 Hz), 131.66 – 131.64 (d, *J* = 2.8 Hz), 131.36 – 130.73 (d, *J* = 95.1 Hz), 131.26, 131.14 – 131.08 (d, *J* = 10.0 Hz), 130.66 – 130.02 (d, *J* = 96.4 Hz), 129.20, 128.87 – 128.79 (d, *J* = 12.3 Hz), 128.28 – 128.20 (d, *J* = 12.4 Hz), 127.12 – 127.04 (d, *J* = 12.6 Hz), 121.97, 121.17.

Diastereotopic splitting was observed for Ph carbons.

HRMS (ESI^+) Calculated for $C_{24}H_{19}O_2PNa^+$ $[M+Na]^+$ 393.1005 found 393.1015.

S2.3 Synthesis and characterization of 1b

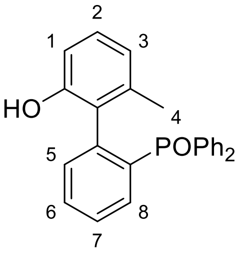

1b

Compound **1b** was synthesised according to previously reported procedures.[S1]

^{1}H NMR ($CDCl_3$, 600 MHz, 295 K): δ 8.66 (brs, 1H, OH), 7.92 – 7.85 (m, 2H, $H_{7,8}$), 7.63 – 7.55 (m, 2H, H_{Ar}), 7.55 – 7.51 (m, 2H, $H_{5,6}$), 7.46 – 7.40 (m, 2H, H_{Ar}), 7.35 (m, 2H, H_{Ar}), 7.33 – 7.27 (m, 1H, H_{Ar}), 7.25 – 7.21 (m, 3H, H_{Ar}), 7.00 – 6.90 (m, 2H, $H_{1,2}$), 6.38 – 6.34 (m, 1H, H_3), 1.49 (s, 3H, H_4).

^{31}P{^{1}H} NMR ($CDCl_3$, 400 MHz, 295 K): δ 30.35.

^{13}C{^{1}H} NMR ($CDCl_3$, 151 MHz, 295 K): δ 154.56, 143.04 – 142.99 (d, *J* = 7.5 Hz), 136.92, 133.29 – 133.23 (d, *J* = 9.9 Hz), 132.94 – 132.86 (d, *J* = 12.7 Hz), 132.69 – 132.67 (d, *J* = 2.4 Hz), 132.49, 132.41 – 131.81 (d, *J* = 90.6 Hz), 132.35 – 132.33 (d, *J* = 2.8 Hz), 131.81, 131.55 – 131.53 (d, *J* = 2.9 Hz), 130.76 – 130.69 (d, *J* = 10.1 Hz), 130.78 – 130.07 (d, *J* = 107.2 Hz), 130.51 – 129.82 (d, *J* = 104.2 Hz), 128.97, 128.88 – 128.80 (d, *J* = 12.0 Hz), 128.18 – 128.10 (d, *J* = 12.6 Hz), 127.08 – 127.00 (d, *J* = 12.6 Hz), 123.27, 119.72, 21.01.

Diastereotopic splitting was observed for Ph carbons.

HRMS (ESI^+) Calculated for $C_{25}H_{21}O_2PNa^+$ $[M+Na]^+$ 407.1164 found 407.1171.

S2.4 Synthesis and characterization of 1'a

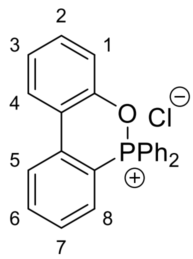

1'a

Compound **1'a** was synthesized as described in Section S4.1.

^{1}H NMR ($CDCl_3$, 600 MHz, 295 K): δ 8.28 (dd, J = 8.2, 5.0 Hz, 1H, H_8), 8.10 – 8.06 (m, 2H, $H_{1,7}$), 7.95 – 7.90 (m, 2H, H_{Ar}), 7.90 – 7.83 (m, 6H, $H_{5,6,Ar}$), 7.81 – 7.76 (m, 4H, H_{Ar}), 7.53 – 7.49 (m, 1H, H_3), 7.46 – 7.40 (m, 2H, $H_{2,4}$).

^{31}P{^{1}H} NMR ($CDCl_3$, 400 MHz, 295 K): δ 59.36.

^{13}C{^{1}H} NMR ($CDCl_3$, 151 MHz, 295 K): δ 146.46 – 146.40 (d, *J* = 8.6 Hz), 137.48, 137.46 – 137.44 (d, *J* = 4.5 Hz), 136.49 – 136.45 (d, *J* = 6.0 Hz), 134.08 – 133.99 (d, *J* = 13.0 Hz), 133.26 – 133.17 (d, *J* = 13.9 Hz), 132.51, 130.94 – 130.84 (d, *J* = 14.7 Hz), 130.82 – 130.72 (d, *J* = 14.3 Hz), 127.47, 126.46, 125.70 – 125.64 (d, *J* = 9.4 Hz), 121.58 – 121.50 (d, *J* = 11.9 Hz), 120.60 – 120.55 (d, *J* = 6.7 Hz), 117.62 – 116.90 (d, *J* = 107.9 Hz), 112.24 – 111.56 (d, *J* = 102.1 Hz).

MALDI: 353.11 ($C_{24}H_{18}OP^+$ $[M]^+$).

S2.5 Synthesis and characterization of 1'b

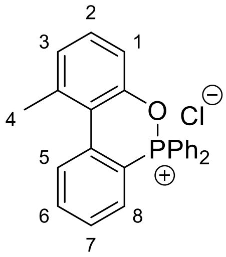

1'b

Compound **1'b** was synthesized as described in Section S4.1.

^{1}H NMR ($CDCl_3$, 600 MHz, 295 K): δ 8.12 (dd, J = 8.2, 5.1 Hz, 1H, H_8), 8.06 – 8.02 (m, 1H, H_7), 7.94 – 7.90 (m, 2H, H_{Ar}), 7.87 – 7.75 (m, 11H, $H_{1,5,6,Ar}$), 7.36 – 7.32 (m, 1H, H_2), 7.25 (s, 1H, H_2), 2.72 (s, 3H, H_4).

^{31}P{^{1}H} NMR ($CDCl_3$, 400 MHz, 295 K): δ 60.16.

^{13}C{^{1}H} NMR ($CDCl_3$, 151 MHz, 295 K): δ 146.79 – 146.73 (d, *J* = 8.4 Hz), 138.17, 137.47 – 137.46 (d, *J* = 2.9 Hz), 136.65 – 136.62 (d, *J* = 4.9 Hz), 136.25 – 136.23 (d, *J* = 2.6 Hz), 134.17 – 134.09 (d, *J* = 12.7 Hz), 133.51 – 133.42 (d, *J* = 14.3 Hz), 131.19 – 131.04 (d, *J* = 23.0 Hz), 130.77 – 130.68 (d, *J* = 14.1 Hz), 129.99, 129.89, 129.88 – 129.82 (d, *J* = 9.1 Hz), 123.00 – 122.92 (d, *J* = 11.5 Hz), 118.07 – 118.03 (d, *J* = 6.4 Hz), 116.85 – 116.14 (d, *J* = 107.3 Hz), 116.04 – 115.35 (d, *J* = 103.9 Hz), 23.40.

MALDI: 367.13 ($C_{25}H_{20}OP^+$ $[M]^+$).

S3. Chiral HPLC analysis of 1a and 1b

Chiral stationary phase high-performance liquid chromatography (chiral HPLC) analysis was performed to analyse **1a** and **1b** (see Figure S1) on a diacel ChiralPak IF column (4.6 mm × 25 mm, 5 µm particle size). A mixture of *i*-PrOH:*n*-hexane (15:85 *v*/*v*) was used as an eluent at 25 °C with a flowrate of 2 mL min^{-1}. Traces based on the absorbance at 240 nm are reported. HPLC data was analysed in Open Labs CDS software, and traces were exported as .csv data files for further plotting in OriginPro 2021b.

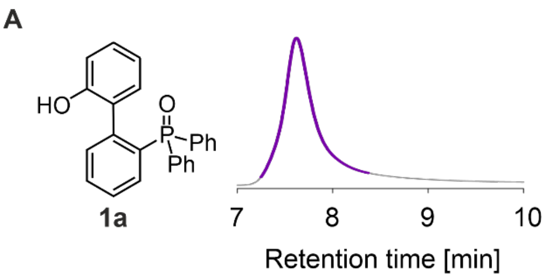

Figure S1: Chiral HPLC analysis of 1a and 1b. Conditions: ChiralPak IF column (4.6 mm × 25 mm, 5 µm particle size), 25 °C, *i*-PrOH:*n*-hexane (15:85 *v*/*v*), 2 mL min^{-1}. A Chemical structure (*left*) and normalized chiral HPLC trace (*right*) of 1a. No atropisomers were observed but a single peak, highlighted in purple. B Chemical structure (*left*) and normalized chiral HPLC trace (*right*) of 1b. Two atropisomeric peaks were observed as highlighted in blue and red.

S4. Motor operations

S4.1 General procedure

To a stock solution of **1b** or **1a** in $CDCl_3$ [**1**] = 5.0 mM was added $SOCl_2$, so that [$SOCl_2$] = 50 mM, at room temperature. After completion of the transformation to **1'** (as indicated by ^{1}H NMR spectroscopy, typically 1 h) nucleophilic displacement was initiated by addition of an alcohol ROH, so that [ROH] = 50 mM or water H_2O, so that [H_2O] = 50 mM. The reaction completed rapidly (<5 min), as indicated by ^{1}H NMR spectroscopy.

S4.2 ^1H and $^{31}P\{^1H\}$ NMR spectra of closing 1b followed by H_2O hydrolysis

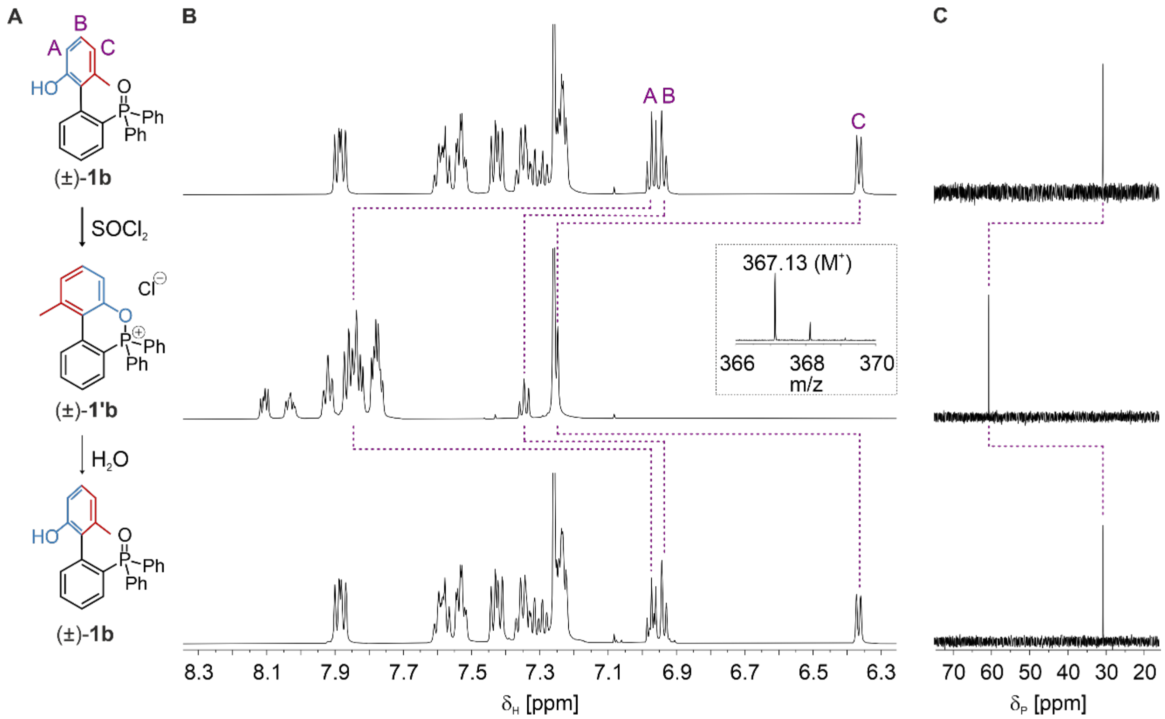

Figure S2: Chemical transformation of 1b upon treatment with $SOCl_2$ followed by H_2O in $CDCl_3$ at room temperature showing the formation and hydrolysis of the oxyphosphonium salt 1'b. A Reaction scheme for the chemical transitions. B Partial ^1H NMR (600 MHz, $CDCl_3$, 293 K) spectra of 1b (*top*), 1 hour following the addition of $SOCl_2$ to form 1'b (*middle*), and 5 minutes following the subsequent addition of H_2O to reform 1b (*bottom*). Changes in the chemical shifts of protons A, B, C and D are indicated by dashed lines. Insert shows MALDI-ToF MS spectrum of 1'b. C Partial $^{31}P\{^1H\}$ NMR (126 MHz, $CDCl_3$, 293 K) spectra of 1b (*top*), 1 hour following the addition of $SOCl_2$ to form 1'b (*middle*), and 5 minutes following the subsequent addition of H_2O to reform 1b (*bottom*).

S4.3 Chemically-driven racemisation of (+)- and (−)-1b

Atropisomers of **1b** were separated by preparative chiral HPLC (ChiralPak IF column (4.6 mm × 25 mm, 5 μm particle size), 25 °C, *i*-PrOH:*n*-hexane (15:85 *v*/*v*), 2 mL min^{-1}), the right peak was labelled (highlighted in red) as (−)-**1b**, and the left peak (highlighted in blue) as (+)-**1b** (see Figure S3).

Pure, separated samples of (+)- and (−)-**1b** were dissolved in $CDCl_3$, so that [**1b**] = 5.0 mM and were operated according to the general procedure given in S4.1. HPLC analysis (ChiralPak IF column (4.6 mm x 25 mm, 5 μm particle size), 25 °C, *i*-PrOH:*n*-hexane (15:85 *v*/*v*), 2 mL min^{-1}) of the operated samples revealed complete racemization (see Figure S3).

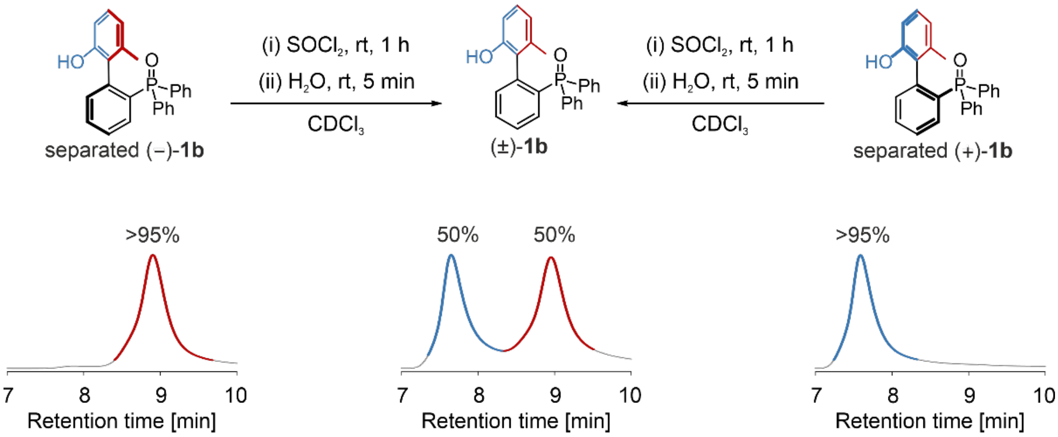

Figure S3: Chemically driven racemization of separated enantiomers of 1b. Treatment of chiral HPLC separated enantiomers of 1b with $SOCl_2$ followed by H_2O (*top*) resulted in complete racemization as determined by chiral HPLC (*bottom*, ChiralPak IF column (4.6 mm x 25 mm, 5 μm particle size), 25 °C, *i*-PrOH:*n*-hexane (15:85 *v*/*v*), 2 mL min^{-1}).

S4.4 Directional opening of 1'b.

To a stock solution of **1b** in $CDCl_3$ [**1**] = 5.0 mM was added $SOCl_2$, so that [$SOCl_2$] = 50 mM, at room temperature. After completion of the transformation to **1'b**, the enantioselective Appel reaction (as shown in Figure S8C) was initially screened by the addition of different commercially available chiral alcohols, so that [alcohol] = 50 mM] under different conditions, as given in Table 1. The reaction completed rapidly (<5 min), as indicated by ^{1}H NMR spectroscopy. The samples were analysed by HPLC (ChiralPak IF column (4.6 mm x 25 mm, 5 µm particle size), 25 °C, CH_2Cl_2:*i*-PrOH:CF_3CO_2H (94:5:1, *v*/*v*/*v*):*n*-hexane (35:65, *v*/*v*), 2 mL min^{-1}).

Table S1: Screening of alcohols and solvents towards the enantioselective opening of **1'b**.

Alcohol	Solvent	e.e.%
**(*R*)/(*S*)-2,2,2-Trifluoro-1-(9-anthryl)ethanol**	Chloroform	0
**(*R*)/(*S*)-1-Phenyl-1-propanol**	Chloroform	2.4
**(*R*)/(*S*)-12-(1-Naphtyl)ethanol**	Chloroform	3.8
**(+)/(-)-Borneol**	Chloroform	2.4
**(+)/(-)-Terpinen-4-ol**	Chloroform	1.5
**(*R*)/(*S*)-1-(4-Methoxyphenyl)ethanol**	Chloroform	0.5
**(*R*)/(*S*)-1-Phenylethan-1-ol**	Chloroform	11
**(*R*)/(*S*)-1-Phenylethan-1-ol**	THF[a]	4.8
**(*R*)/(*S*)-1-Phenylethan-1-ol**	Toluene[a]	4.3
**(*R*)/(*S*)-1-Phenylethan-1-ol**	Dichloromethane[a]	8.3
**(*R*)/(*S*)-1-Phenylethan-1-ol**	Diethyl ether[a, b]	0
**(*R*)/(*S*)-1-Phenylethan-1-ol**	Chloroform[c]	24

[a]Closing of **1b** to **1'b** was performed in the stated solvent. [b]No formation of **1'b** was observed, probably due to poor solubility of **1b**. [c]Opening was carried out at 4 °C instead of room temperature.

With the optimized procedure in hands, a 5 mM solution of **1b** (76.9 µg, 0.200 µmol, 1.0 eq.) in $CDCl_3$ (0.4 mL) was treated with $SOCl_2$ (0.145 µL, 2.00 µmol, 10 eq.). After 5 min at room temperature, the reaction was cooled to 4 °C and either (*R*)- or (*S*)-1-phenyl ethan-1-ol (244 µg, 2.00 µmol, 10 eq.) was added. After completion of the reaction (as indicated by ^{1}H NMR spectroscopy, typically 16 h, see Figure S5) at 4 °C, the samples were allowed to warm up to rt and analyzed, without any further workup, by HPLC (ChiralPak IF column (4.6 mm × 25 mm, 5 µm particle size), 25 °C, CH_2Cl_2:*i*-PrOH:CF_3CO_2H (94:5:1, *v*/*v*/*v*):*n*-hexane (35:65, *v*/*v*), 2 mL min^{-1}), revealing an enantiomeric excess (e.e.) of 24% for (−)-**1b** when (*R*)-1-phenylethan-1-ol was used and (+)-**1b** when (*S*)-1-phenyl ethan-1-ol (see Figure 3 and Figure S4).

Enantioenriched samples of (+)- and (−)-**1b**, after operation (and without any further workup), were dried in vacuo and dissolved in toluene. The solutions were heated in a sand bath at 100 °C for 20 h and were injected into chiral HPLC (ChiralPak IF column (4.6 mm × 25 mm, 5 µm particle size),

25 °C, CH_2Cl_2:*i*-PrOH:CF_3CO_2H (94:5:1, *v*/*v*/*v*):*n*-hexane (35:65, *v*/*v*), 2 mL min^{-1}) showing full racemization (see Figure S4).

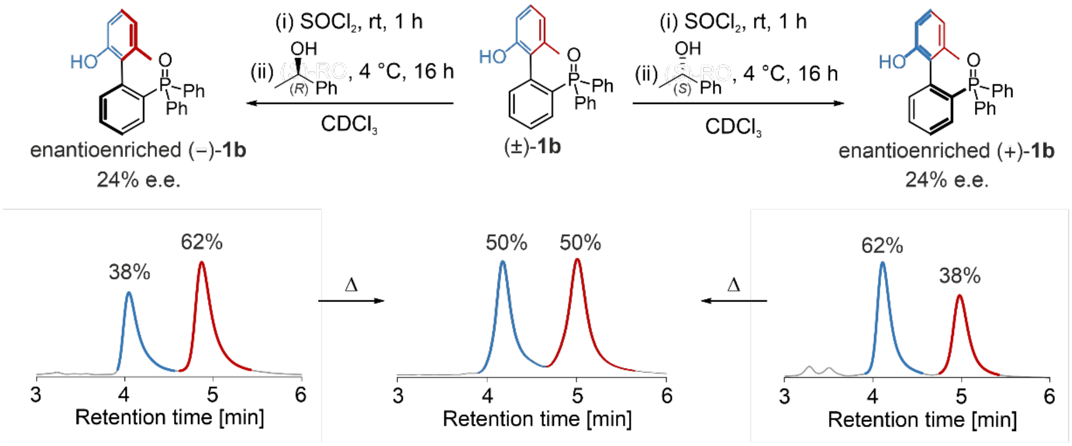

Figure S4: Directional operation of motor molecule 1b. Treatment of racemic 1b with $SOCl_2$ followed by (*R*)-1-phenylethan-1-ol (*left*) or (*S*)-1-phenylethan-1-ol (*right*) results in formation of enantioenriched (±)-1b in 24% e.e. as determined by chiral HPLC (ChiralPak IF column (4.6 mm × 25 mm, 5 µm particle size), 25 °C, CH_2Cl_2:*i*-PrOH:CF_3CO_2H (94:5:1, *v*/*v*/*v*):*n*-hexane (35:65, *v*/*v*), 2 mL min^{-1}). Heating the enantioenriched samples at 100 °C for 100 hours resulted in complete racemization.

S4.5 ^{1}H NMR analysis of directional opening of 1'b

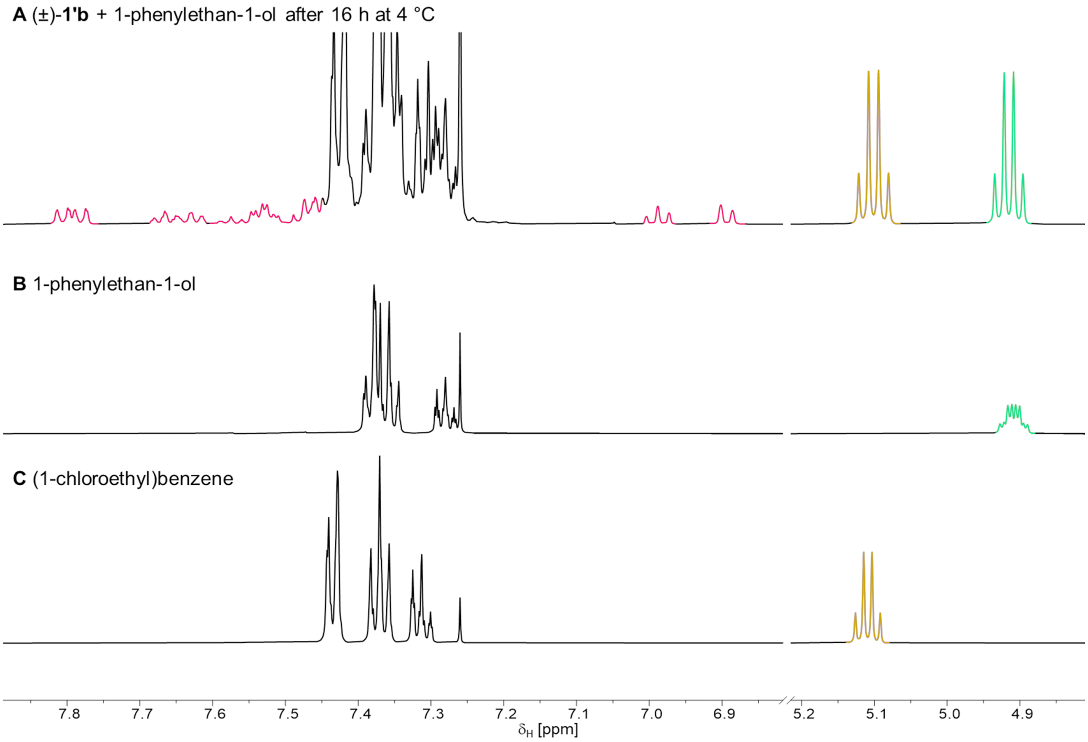

Figure S5: Partial ^{1}H NMR (600 MHz, $CDCl_3$, 293 K) spectra of **A** (±)-**1'b** 16 h after addition of 10 eq. 1-phenylethan-1-ol, showing the re-formation of (±)-**1b** (red) and the conversion of 1-phenylethan-1-ol (green) to (1-chloroethyl)benzene (yellow), **B** 1-phenylethan-1-ol, **C** (1-chloroethyl)benzene.

S4.6 ^{1}H NMR analysis of consecutively operating 1a

The consecutive closing and opening of **1a** was performed according to the procedure in Section S4.4, differing only by the presence of excess NEt_3 to scavenge the resulting HCl. Specifically, a 5 mM solution of **1a** (74.1 µg, 0.200 µmol, 1.0 eq.) in 1% NEt_3 in $CDCl_3$ (0.4 mL) was treated with $SOCl_2$ (0.145 µL, 2.00 µmol, 10 eq.). After 5 min at rt, the reaction was cooled to 4 °C and (*R*)-1-phenyl ethan-1-ol (244 µg, 2.00 µmol, 10 eq.) was added. After completion of the reaction (as indicated by ^{1}H NMR spectroscopy, see Figure S6) at 4 °C, the samples were allowed to warm up to rt and the subsequent addition of 10 eq. $SOCl_2$ and (*R*)-1-phenyl ethan-1-ol was repeated identically for four times. Presence or absence of NEt_3 did not show a difference in face-selectivity of the nucleophilic attack when **1b** was used, leading to the same enatioenrichment of 24% e.e..

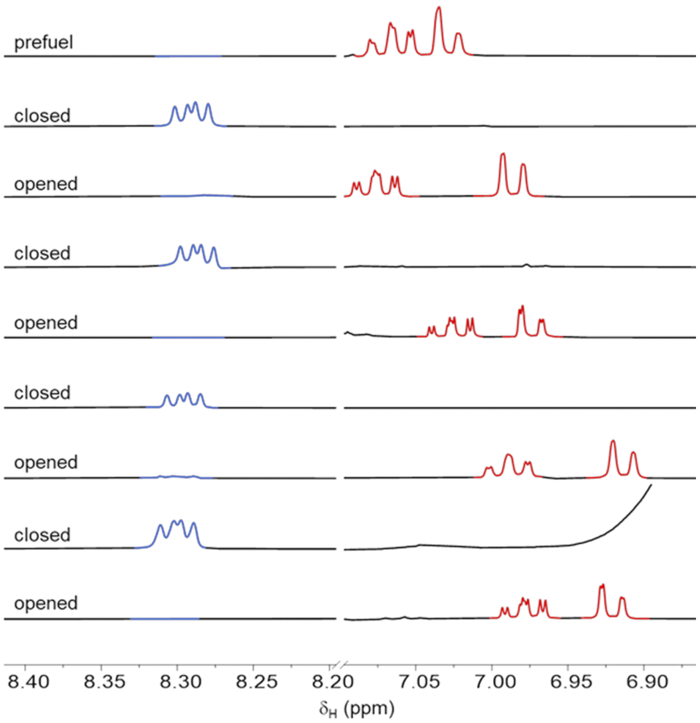

Figure S6: Partial ^{1}H NMR (600 MHz, $CDCl_3$, 293 K) spectra of consecutive operation on **1a**. Separate diagnostic proton signals for **1a** and **1'a** are shown and highlighted in red and blue, respectively. Peak shape and chemical shifts may vary due to changes in the chemical environment in solution over the course of repetitive additions of $SOCl_2$ and (*R*)-1-phenylethan-1-ol.

S4.7 Thermal racemization of 1b

An enantiopure solution of **1b** (1 mM in 1,2-dichlorobenzene) was heated to 130 °C and the racemization was monitored by chiral HPLC (ChiralPak IF column (4.6 mm × 25 mm, 5 μm particle size), 25 °C, *n*-hexane:*i*-PrOH (85:15, *v*/*v*), 2 mL min^{-1}).

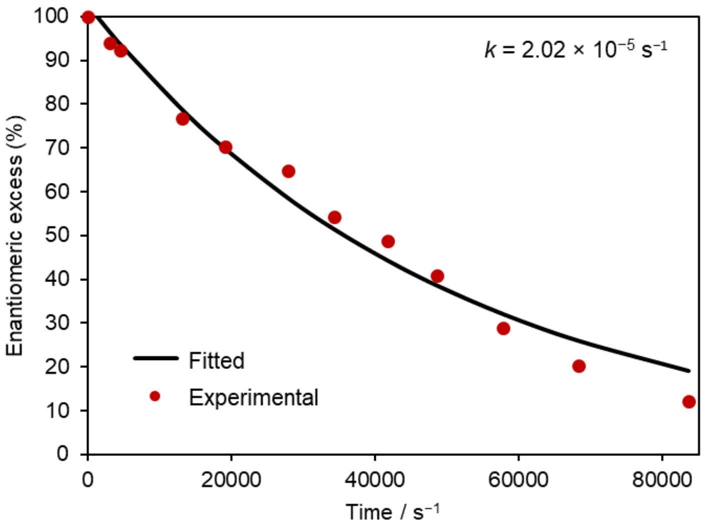

Figure S7: Thermally driven racemization upon heating an enantiopure solution of **1b** (1 mM in 1,2-dichlorobenzene) as monitored by chiral HPLC (ChiralPak IF column (4.6 mm × 25 mm, 5 μm particle size), 25 °C, *n*-hexane:*i*-PrOH (85:15, *v*/*v*), 2 mL min^{-1}). The rate of racemization indicates an energy barrier of ~136 kJ mol^{-1} for this process.

S4.8 Proposed detailed mechanism of chemical transitions

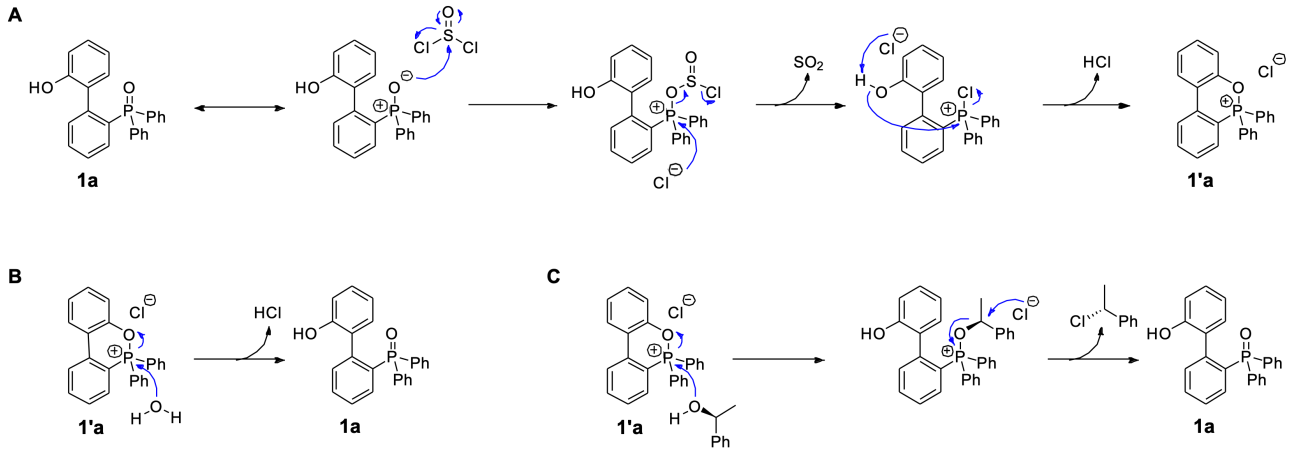

Figure S8: Chemical transitions and proposed mechanisms shown on the example of **1a**. **A** Closing of the intramolecular tether of **1a** by treatment with $SOCl_2$. **B** Opening of tether of **1'a** by hydrolysis upon treatment with H_2O, reforming **1a**. **C** Opening of the tether of **1'a** by attack of the chiral alcohol, followed by substitution of the hydroxyl group by chloride (Appel reaction) reforming **1a**.

S5. Molecular modelling of 1'a and 1'b

The energy minimized molecular structures of **1'a** and **1'b** were calculated using the Merck Molecular Force Field (MMFF) method and are displayed below (Figure S8).

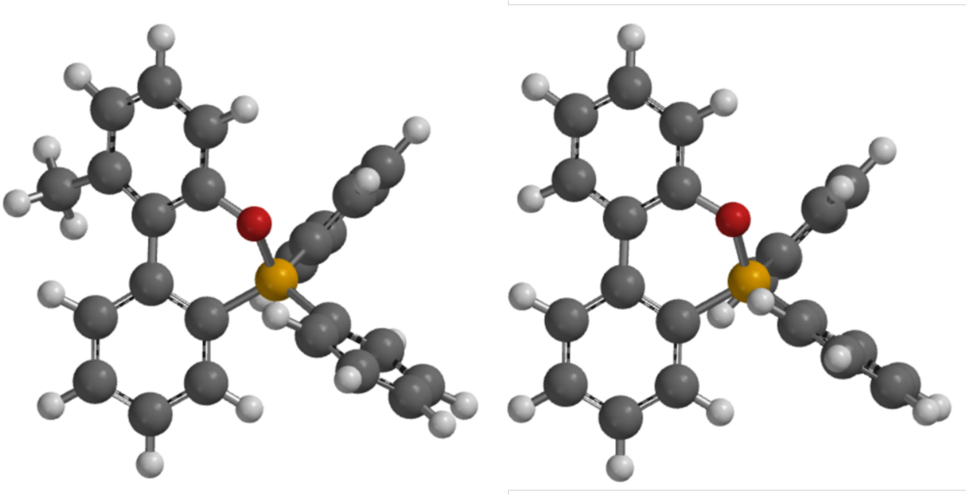

Figure S9: Ball-and-stick representation of MMFF energy minimized structures of **1'a** and **1'b**.

S6. Crystallographic data

X-ray diffraction data for compound **1a** and **1b** were collected using a dual wavelength Rigaku FR-X rotating anode diffractometer using CuKα (λ = 1.54146 Å) radiation, equipped with an AFC-11 4-circle kappa goniometer, VariMAX™ microfocus optics, a Hypix-6000HE detector and an Oxford Cryosystems 800 plus nitrogen flow gas system, at temperatures of 100K and 200K, respectively. Data were collected and reduced using CrysAlisPro v42.[S2] Absorption correction was performed using empirical methods (SCALE3 ABSPACK) based upon symmetry-equivalent reflections combined with measurements at different azimuthal angles. The crystal structure was solved and refined against all F2 values using the SHELX and Olex2 suite of programs.[S3] All atoms were refined anisotropically. Hydrogen atoms were placed in calculated positions and refined using idealized geometries and assigned fixed isotropic displacement parameters.

Table S2 Crystallographic information for compounds **1a** and **1b**

Identification code	1a	1b
Empirical formula	$C_{24}H_{19}O_2P$	$C_{25}H_{21}O_2P$
Formula weight	370.36	384.39
Temperature/K	99.98(10)	199.99(10)
Crystal system	triclinic	monoclinic
Space group	P-1	$P2_1/n$
a/Å	8.7996(4)	10.7395(2)
b/Å	9.7235(4)	12.2076(3)
c/Å	12.1210(4)	15.0330(4)
α/°	106.395(3)	90
β/°	103.569(3)	96.525(2)
γ/°	105.202(4)	90
Volume/$Å^3$	905.19(6)	1958.11(8)
Z	2	4
$\rho_{calc}g/cm^3$	1.359	1.304
μ/mm^{-1}	1.471	1.379
F(000)	388.0	808.0
Crystal size/mm^3	0.03 × 0.02 × 0.01	0.45 × 0.32 × 0.26
Radiation	CuKα (λ = 1.54184)	Cu Kα (λ = 1.54184)
2Θ range for data collection/°	8.064 to 151.828	9.356 to 151.904
Index ranges	-10 ≤ h ≤ 11, -12 ≤ k ≤ 11, -11 ≤ l ≤ 15	-13 ≤ h ≤ 13, -14 ≤ k ≤ 11, -18 ≤ l ≤ 18
Reflections collected	9426	11952
Independent reflections	3606 [R_{int} = 0.0182, R_{sigma} = 0.0233]	3948 [R_{int} = 0.0424, R_{sigma} = 0.0455]
Data/restraints/parameters	3606/0/245	3948/0/254
Goodness-of-fit on F^2	1.066	1.029
Final R indexes [I>=2σ (I)]	R_1 = 0.0341, wR_2 = 0.0886	R_1 = 0.0668, wR_2 = 0.1823
Final R indexes [all data]	R_1 = 0.0362, wR_2 = 0.0899	R_1 = 0.0764, wR_2 = 0.1937
Largest diff. peak/hole / e $Å^{-3}$	0.33/-0.42	0.74/-0.57

CCDC identifiers 2294309 and 2295209 contain the supplementary crystallographic data for this paper. These data can be obtained free of charge via www.ccdc.cam.ac.uk/conts/retrieving.html (or from the Cambridge Crystallographic Data Centre, 12 Union Road, Cambridge CB21EZ, UK; fax:(+44)1223-336-033; or deposit@ccdc.cam.ac.uk).

S7. Spectroscopic data

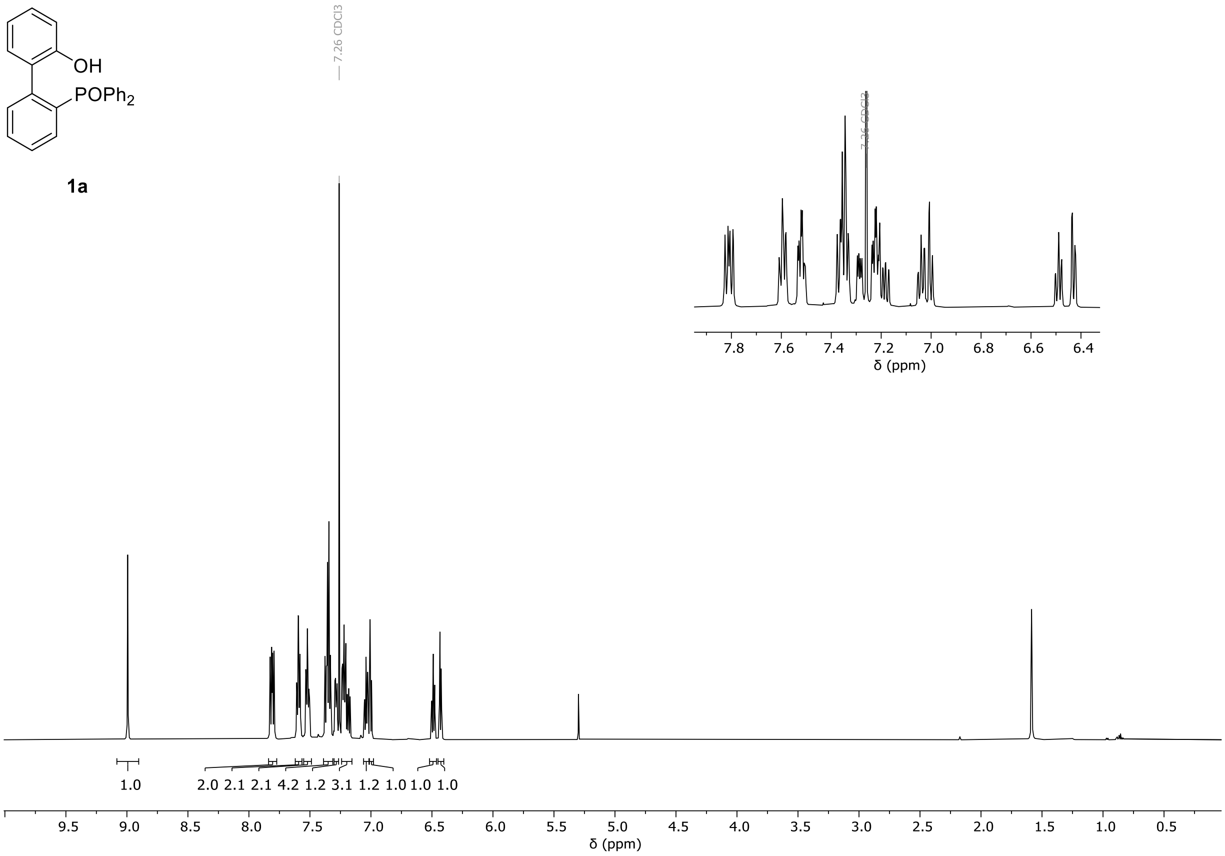

Figure S10. ^{1}H NMR ($CDCl_3$, 600 MHz, 295 K) spectrum of **1a**.

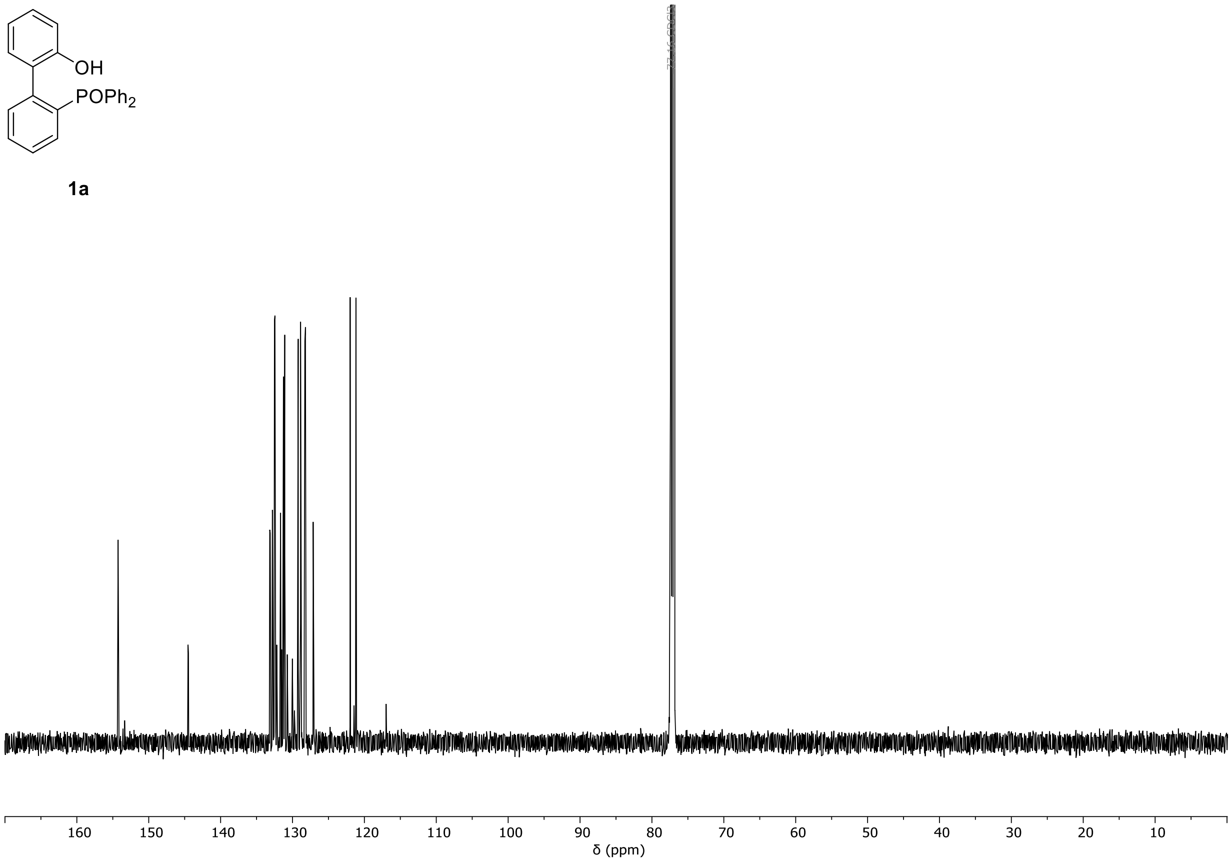

Figure S11. $^{13}C\{^{1}H\}$ NMR ($CDCl_3$, 151 MHz, 295 K) spectrum of **1a**.

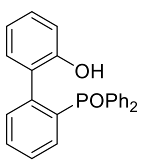

1a

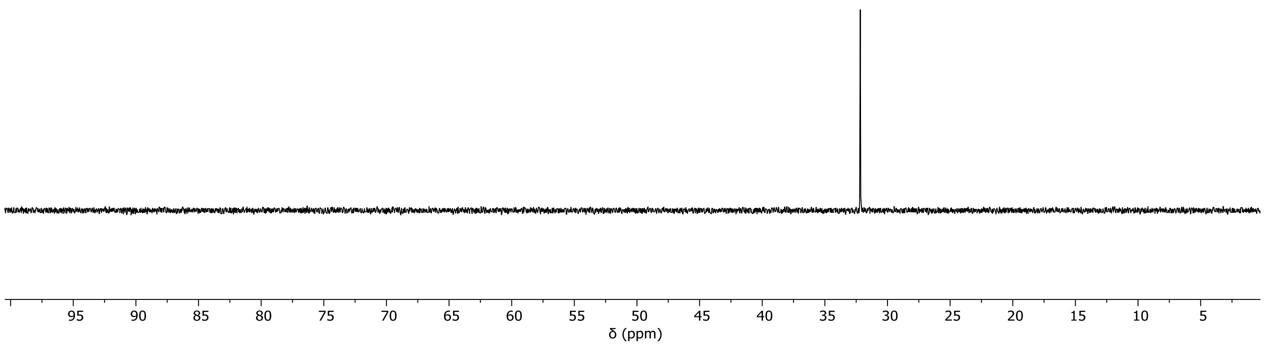

Figure S12. $^{31}P\{1H\}$ NMR ($CDCl_3$, 400 MHz, 295 K) spectrum of **1a**.

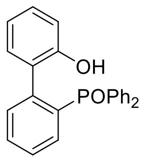

1a

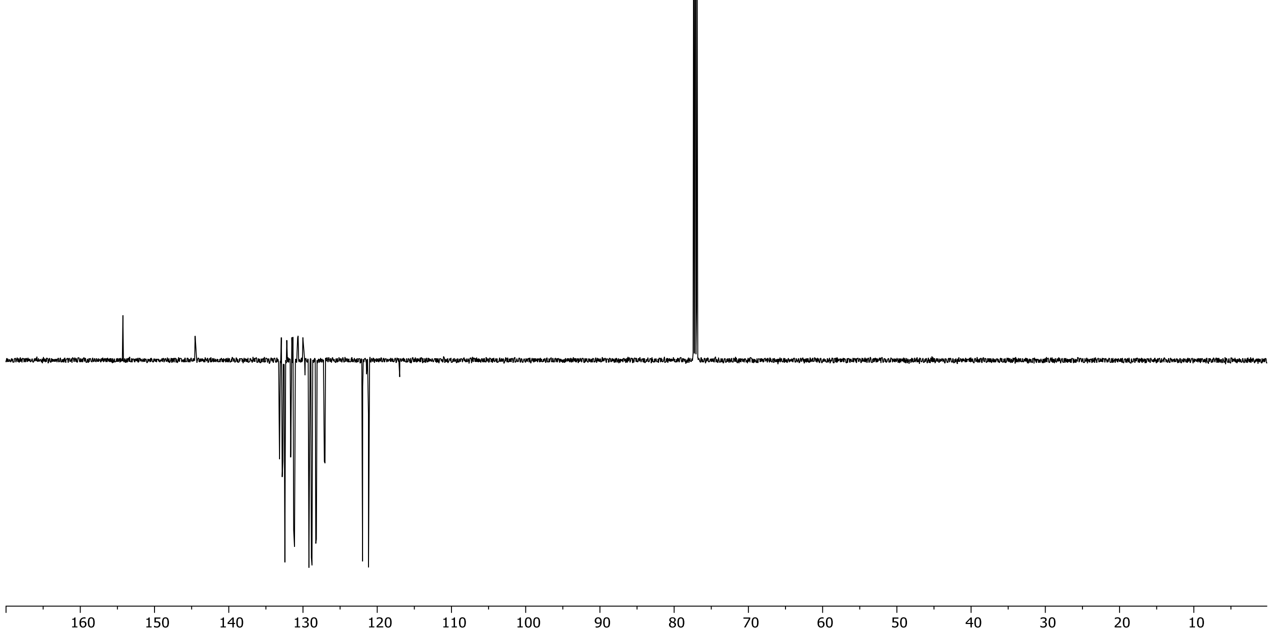

Figure S13. $^{13}C\{^{1}H\}$ DEPTQ NMR ($CDCl_3$, 151 MHz, 295 K) spectrum of **1a**.

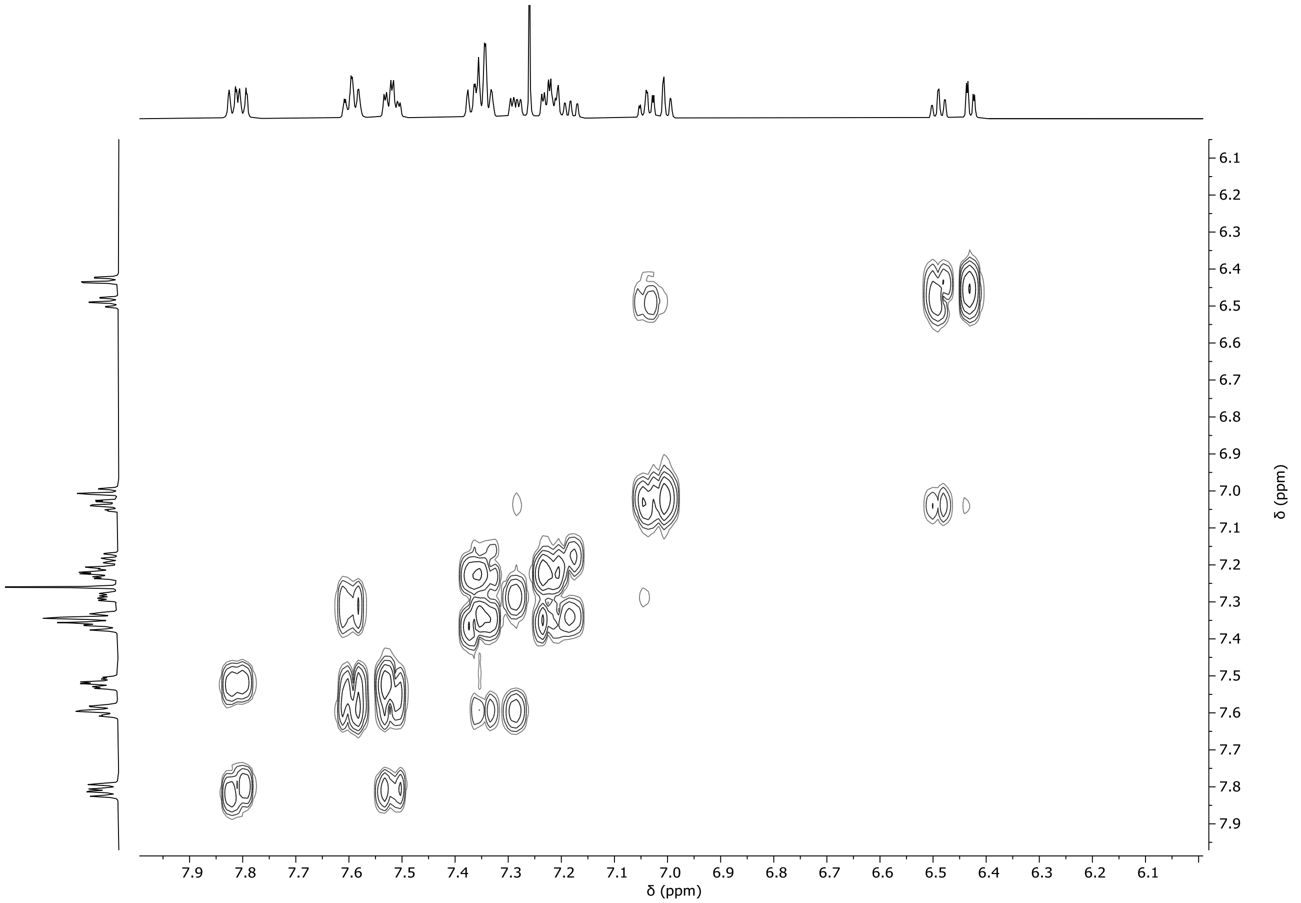

Figure S14. Partial COSY NMR ($CDCl_3$, 600 MHz, 295 K) spectrum of **1a**.

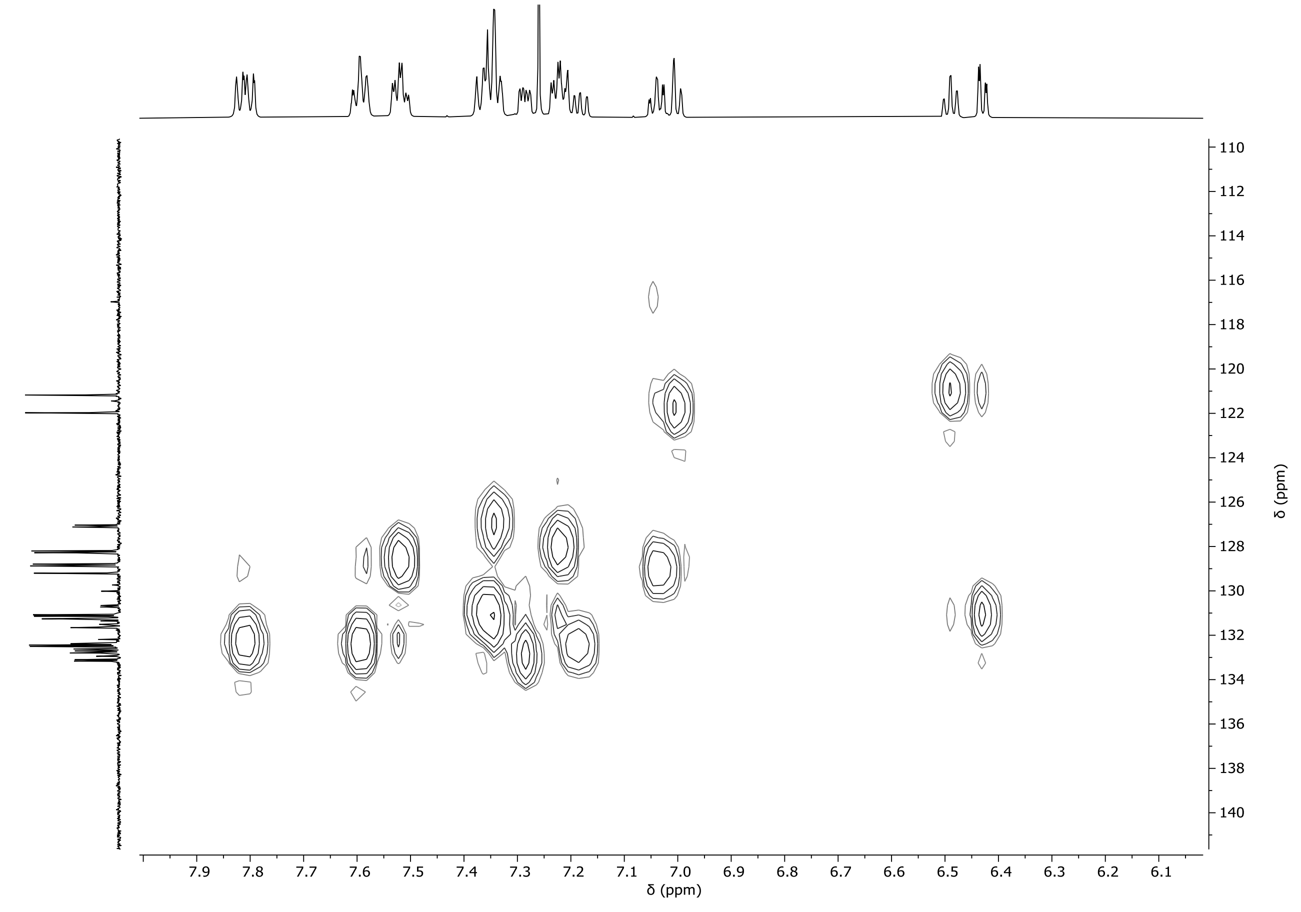

Figure S15. Partial ^{1}H-^{13}C HSQC NMR ($CDCl_3$, 151 MHz, 295 K) spectrum of **1a**.

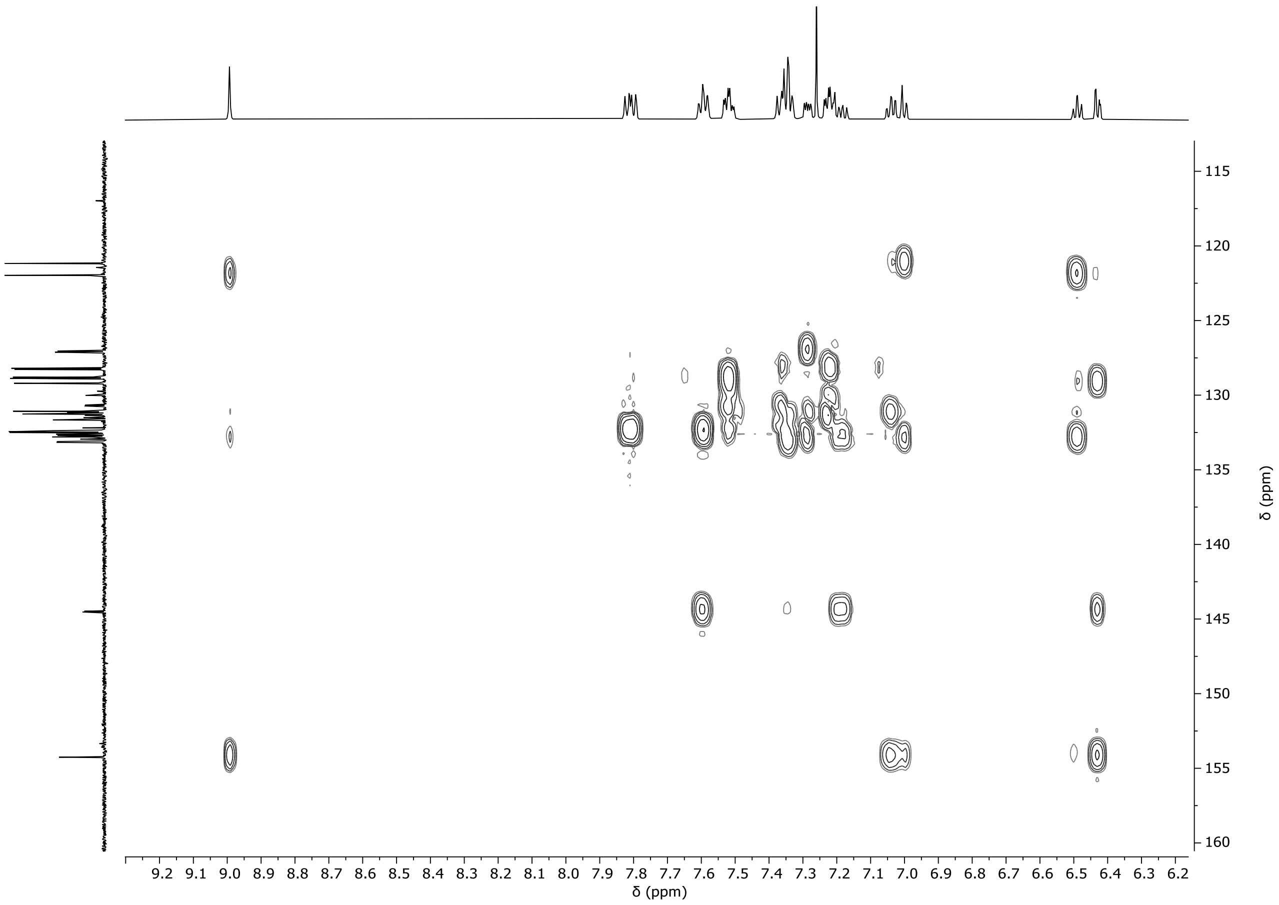

Figure S16. Partial ^{1}H-^{13}C HMBC NMR ($CDCl_3$, 600 MHz, 295 K) spectrum of **1a**.

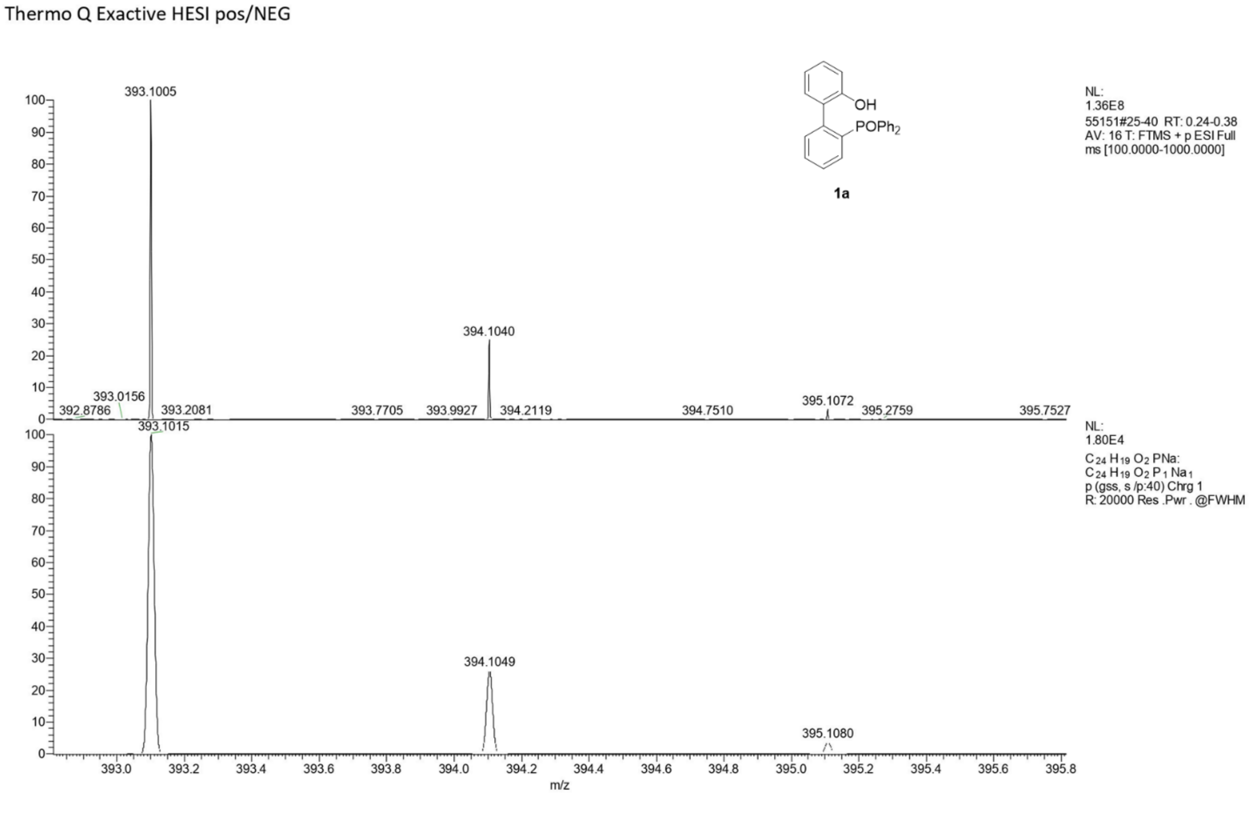

Figure S17. Calculated (*top*) and measured (*bottom*) HRMS (ESI^+) spectrum of **1a** showing $[M+Na]^+$

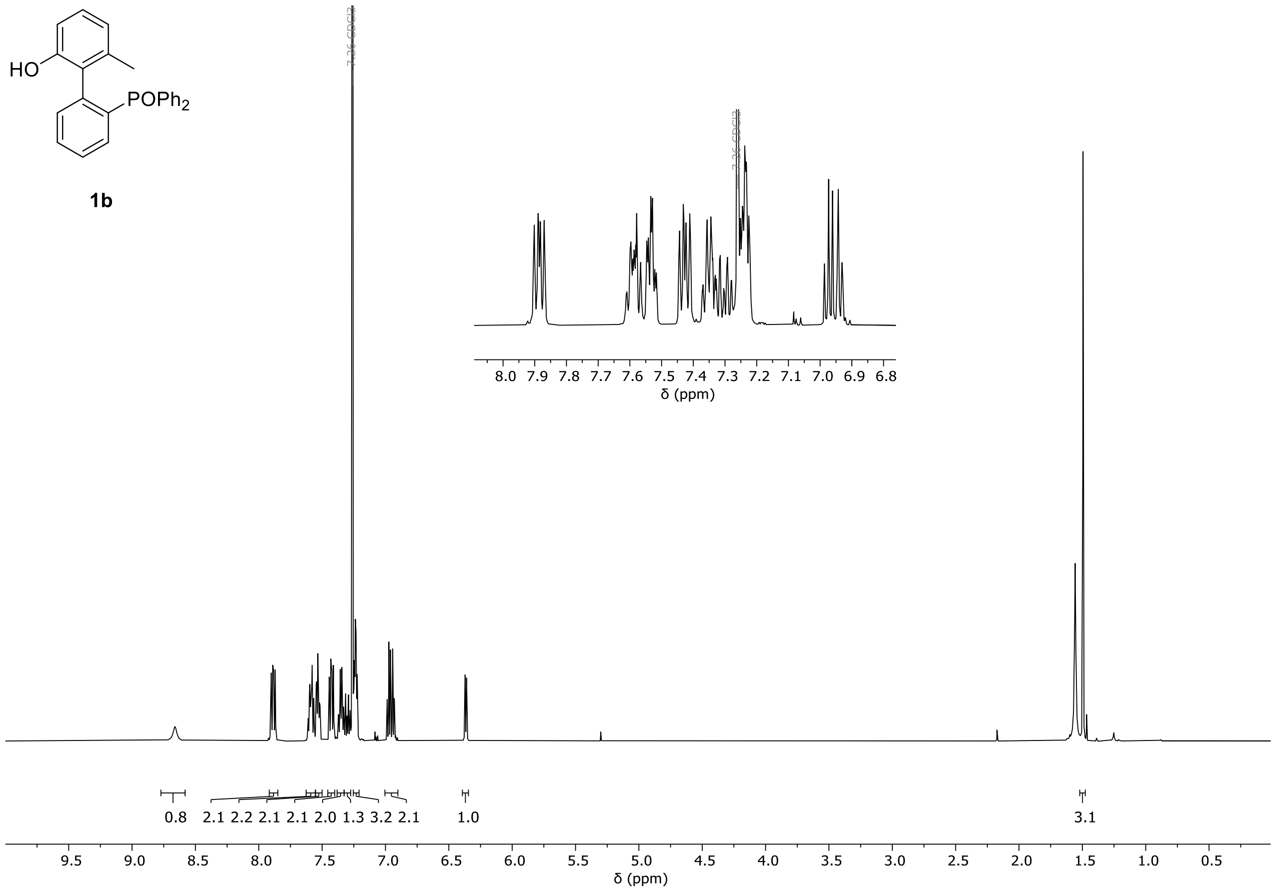

Figure S18. ^{1}H NMR ($CDCl_3$, 600 MHz, 295 K) spectrum of **1b.**

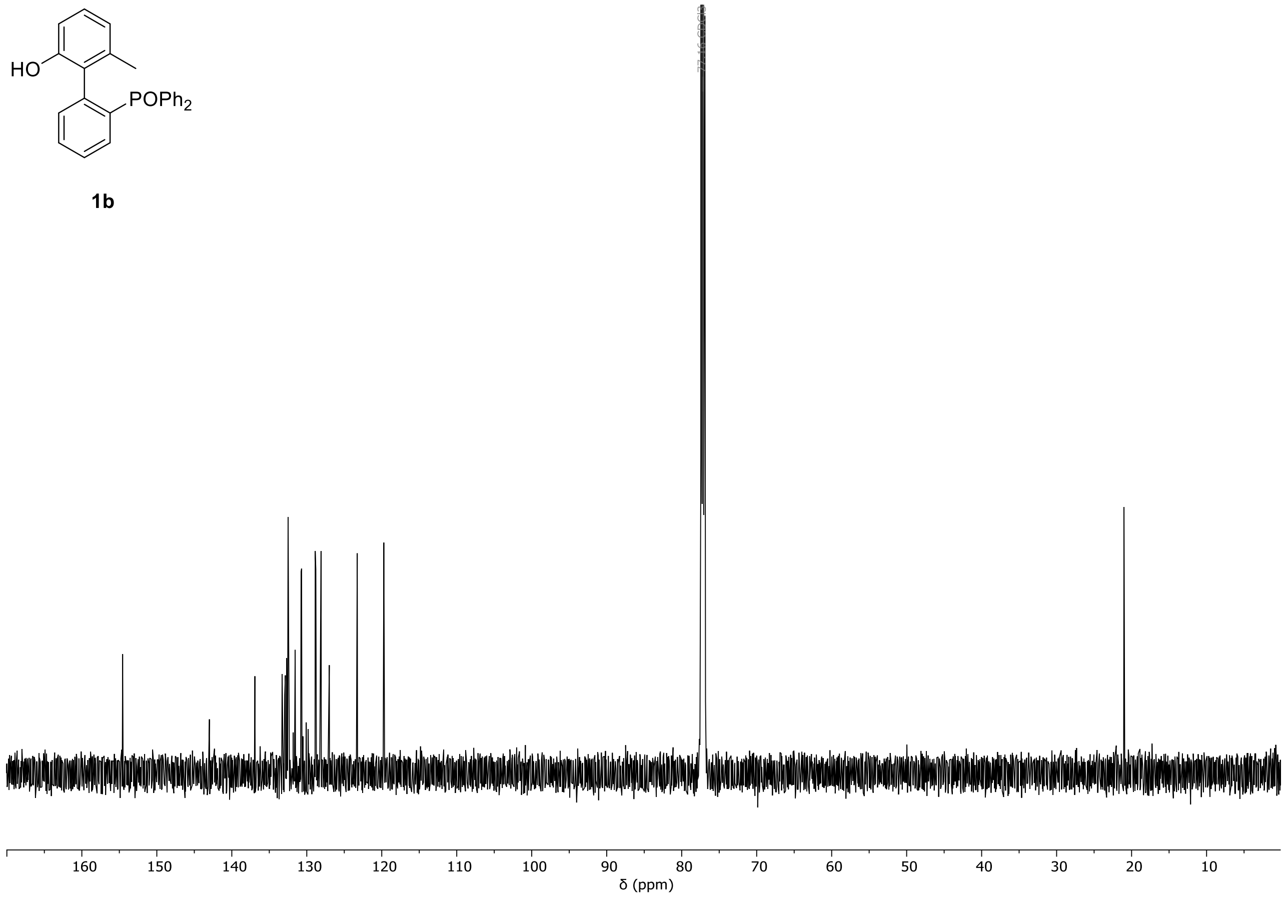

Figure S19. $^{13}C\{^{1}H\}$ NMR ($CDCl_3$, 151 MHz, 295 K) spectrum of **1b**.

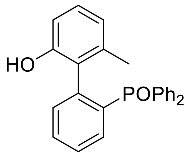

1b

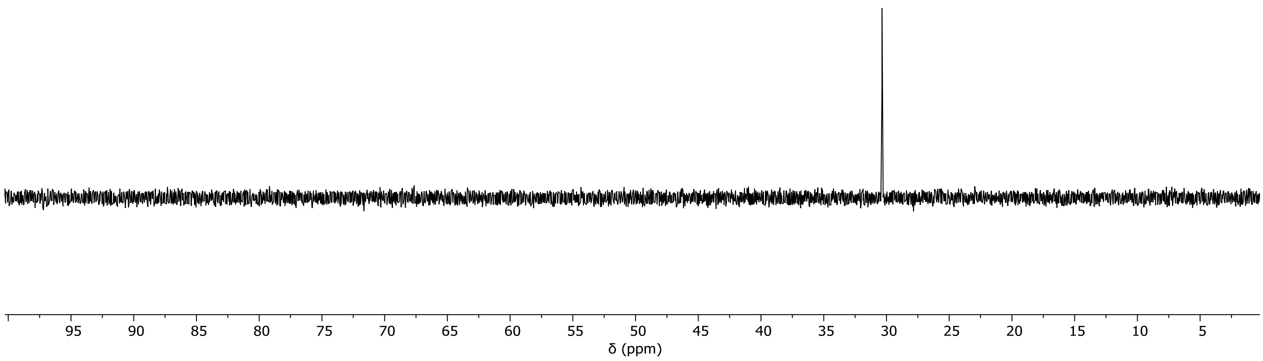

Figure S20. $^{31}P\{^{1}H\}$ NMR ($CDCl_3$, 400 MHz, 295 K) spectrum of **1b**.

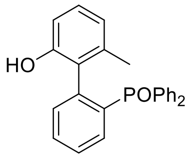

1b

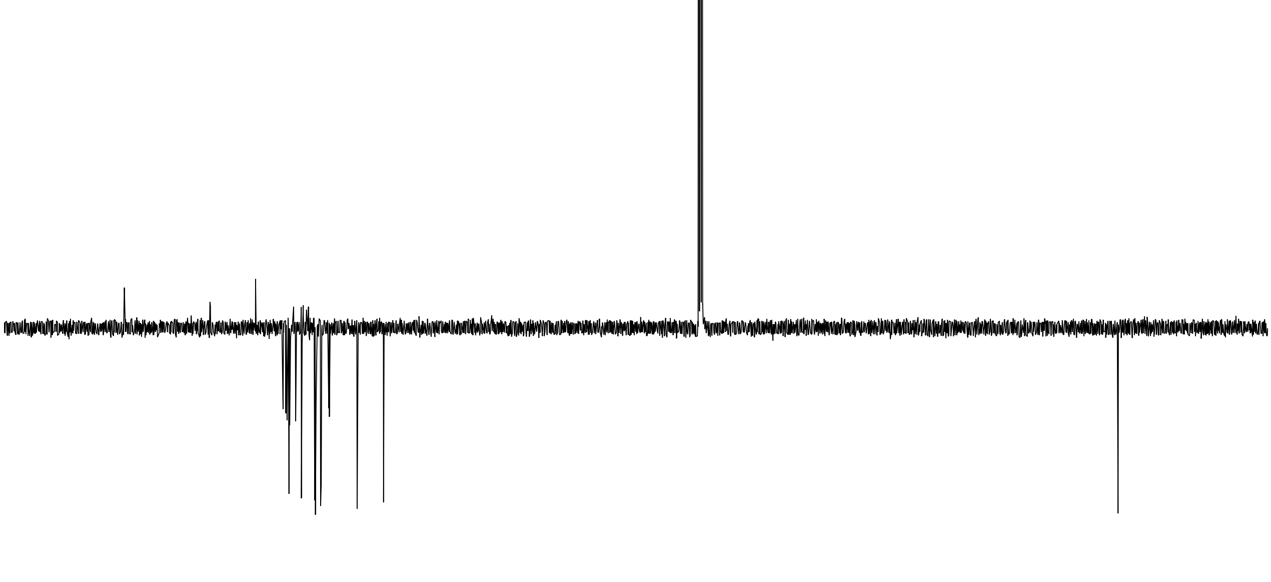

Figure S21. $^{13}C\{^{1}H\}$ DEPTQ NMR ($CDCl_3$, 151 MHz, 295 K) spectrum of **1b**.

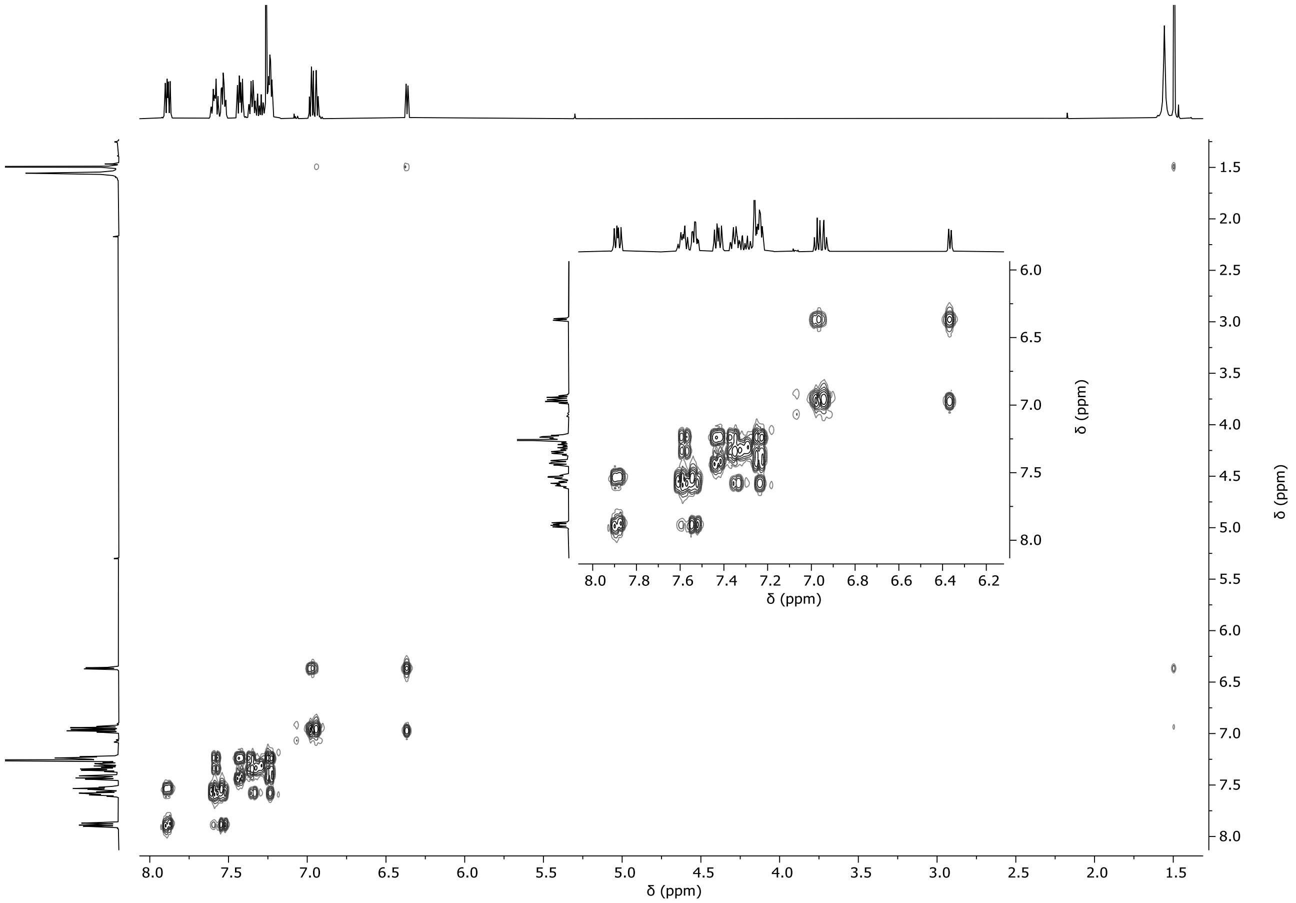

Figure S22. Partial COSY NMR ($CDCl_3$, 600 MHz, 295 K) spectrum of **1b**.

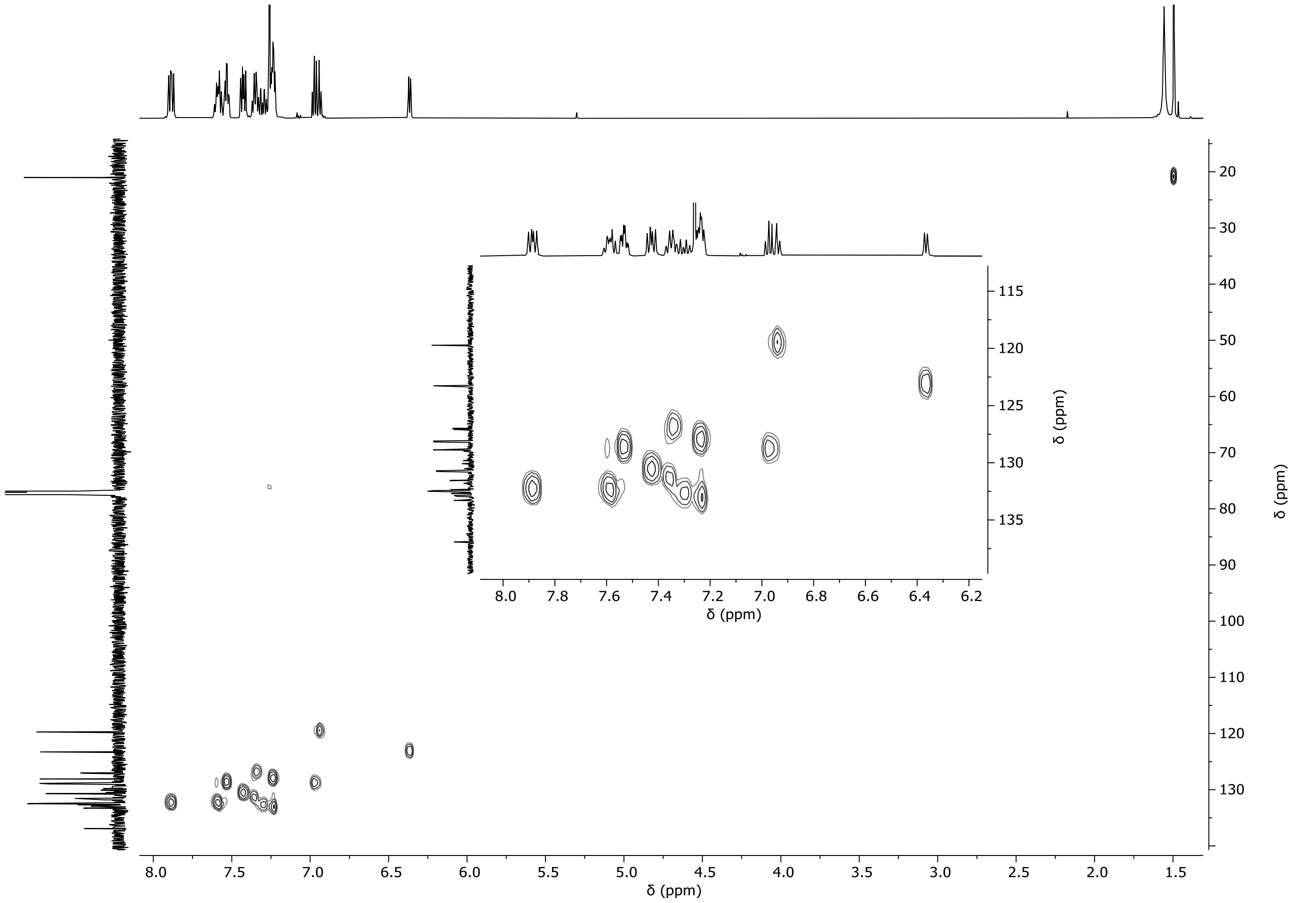

Figure S23. Partial ^{1}H-^{13}C HSQC NMR ($CDCl_3$, 600 MHz, 295 K) spectrum of **1b**.

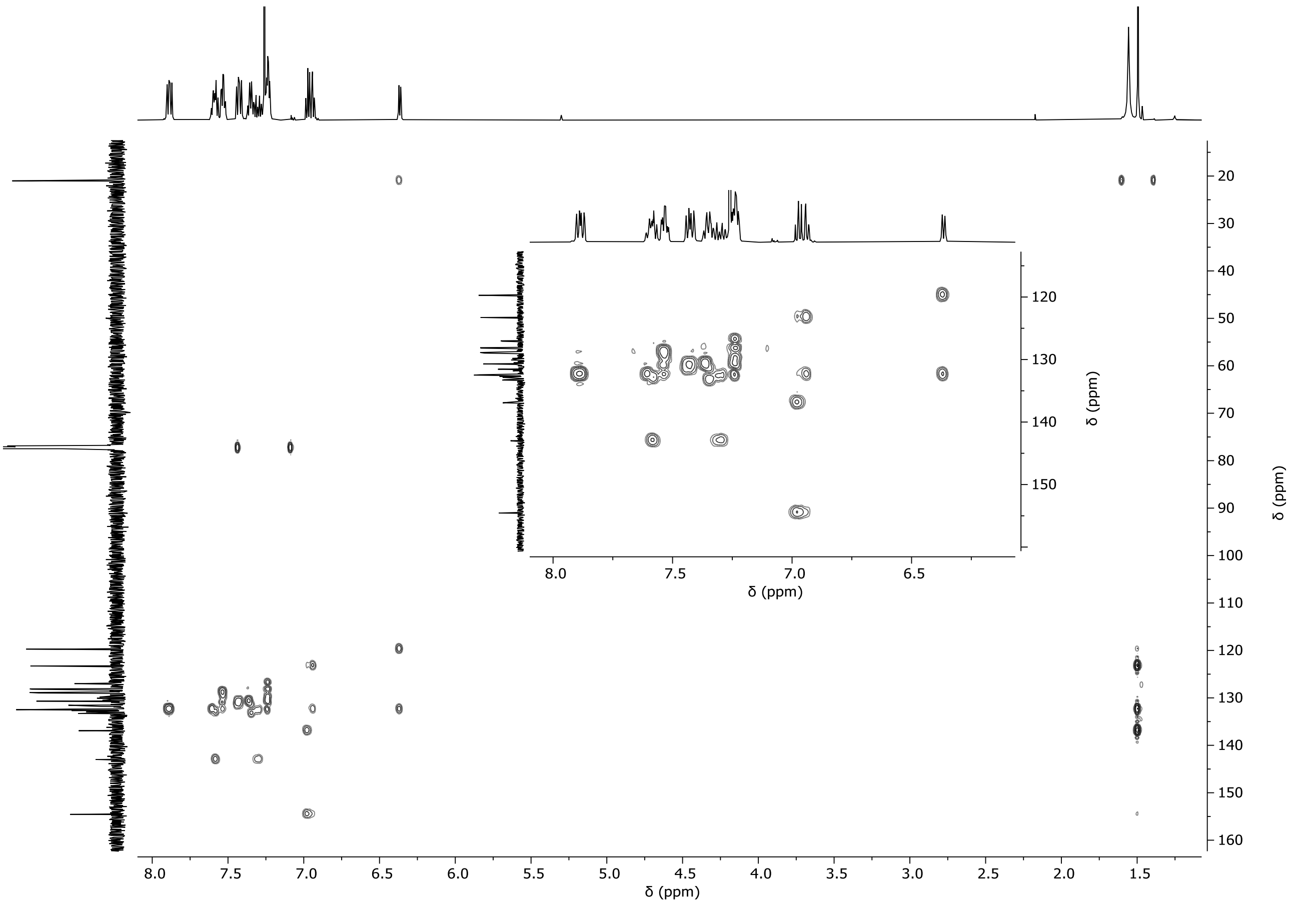

Figure S24. Partial ^{1}H-^{13}C HMBC NMR ($CDCl_3$, 600 MHz, 295 K) spectrum of **1b**.

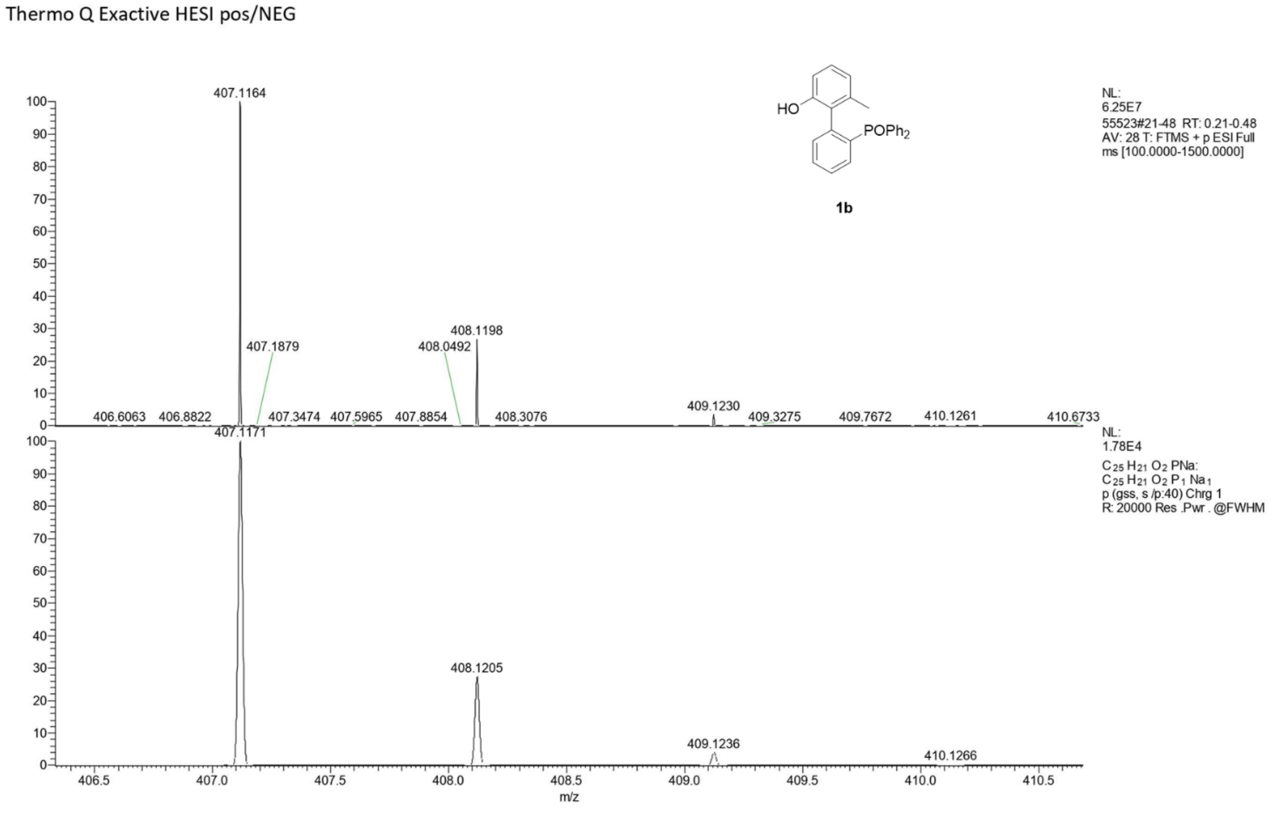

Figure S25. Calculated (*top*) and measured (*bottom*) HRMS (ESI^+) spectrum of **1b** showing $[M+Na]^+$.

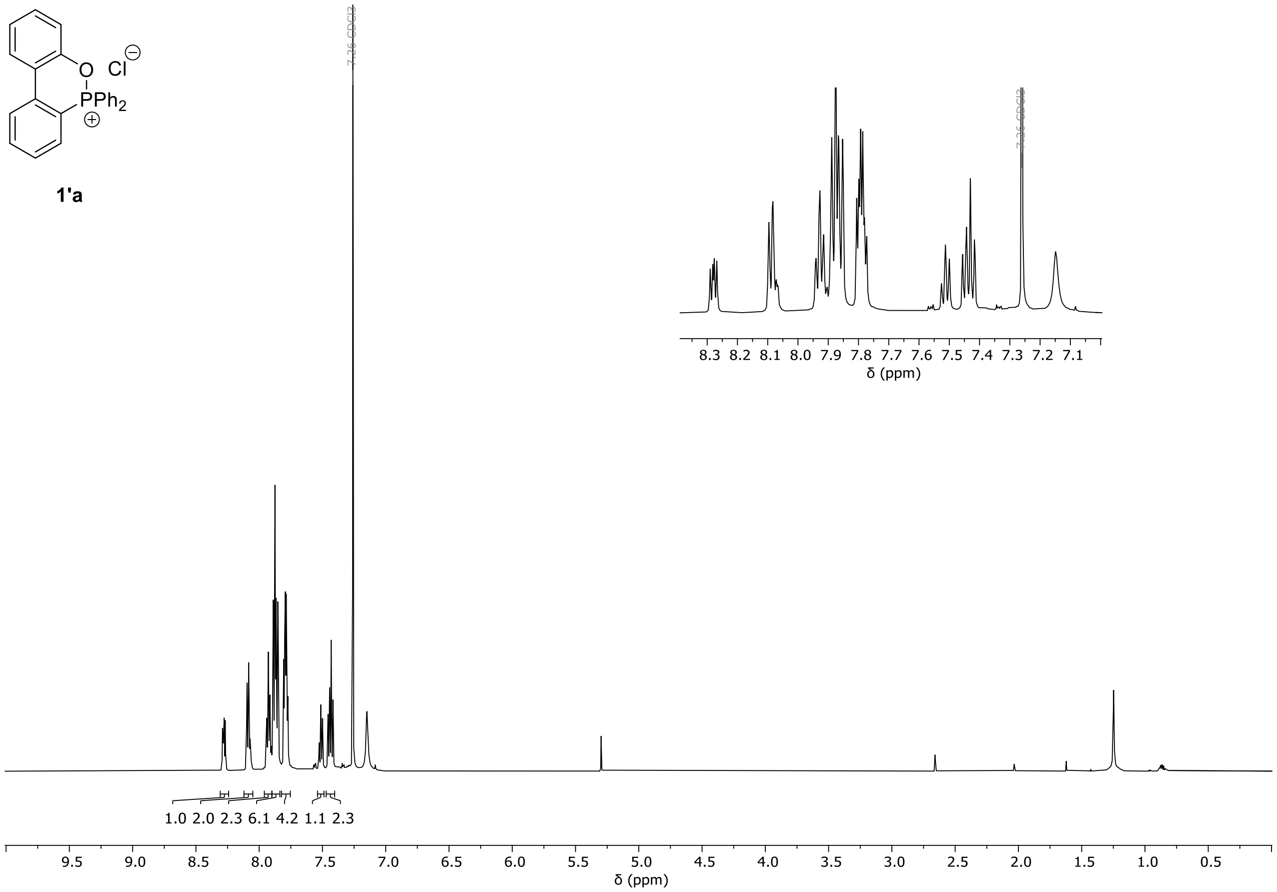

Figure S26. ^{1}H NMR (CDCl$_3$, 600 MHz, 295 K) spectrum of **1′a.**

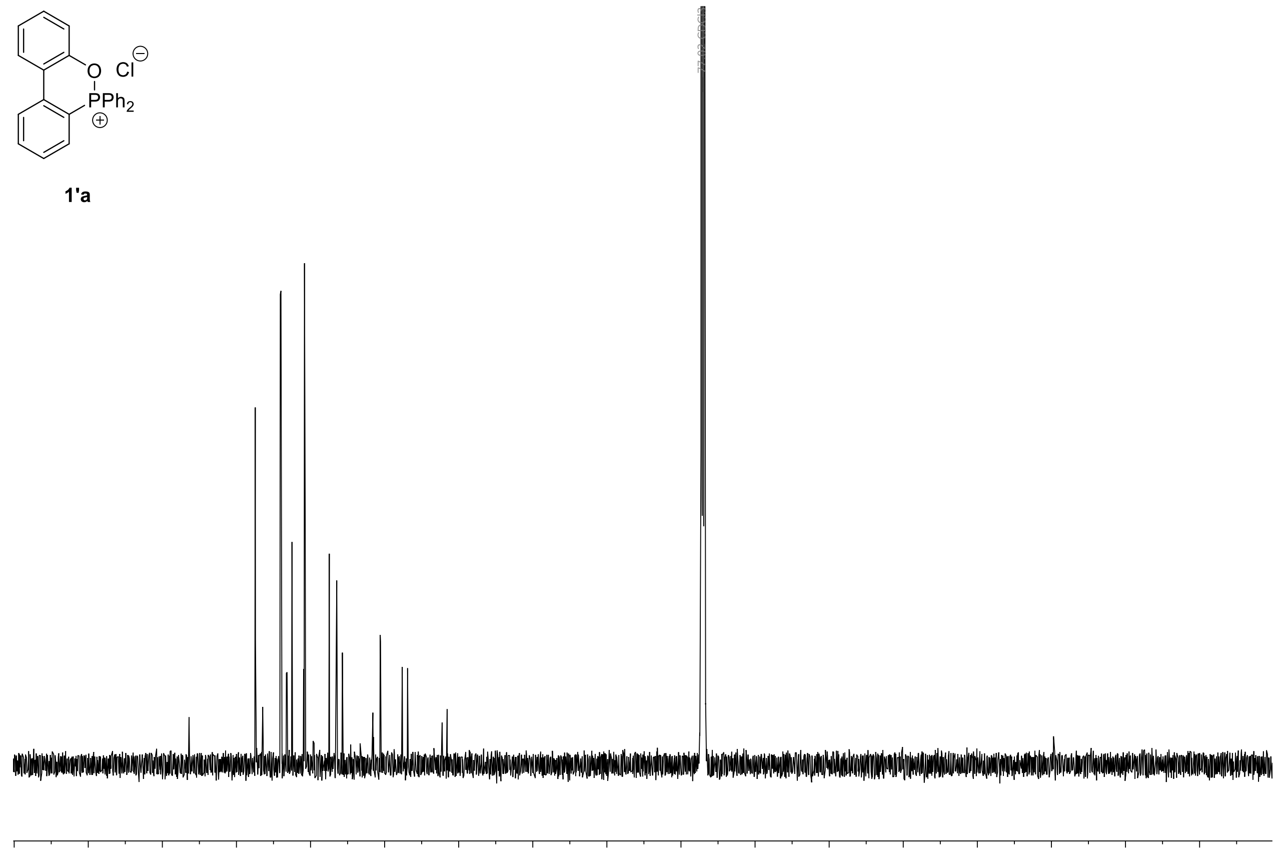

Figure S27. ^{13}C{^{1}H} NMR (CDCl$_3$, 151 MHz, 295 K) spectrum of **1**′**a**.

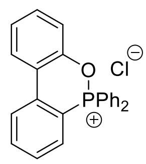

1'a

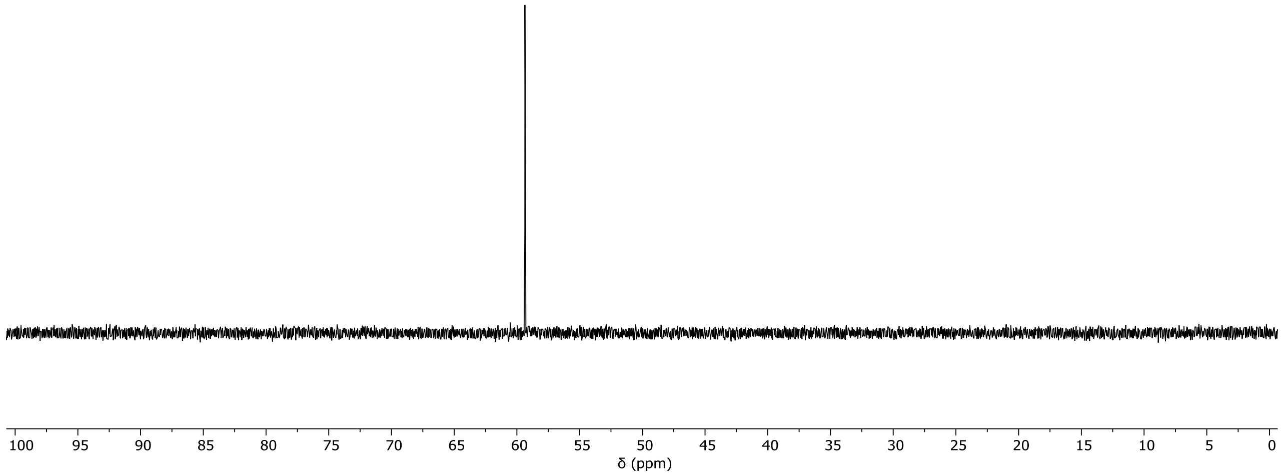

Figure S28. $^{31}P\{^{1}H\}$ NMR ($CDCl_3$, 151 MHz, 295 K) spectrum of **1′a**.

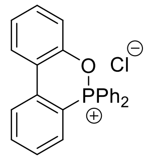

1'a

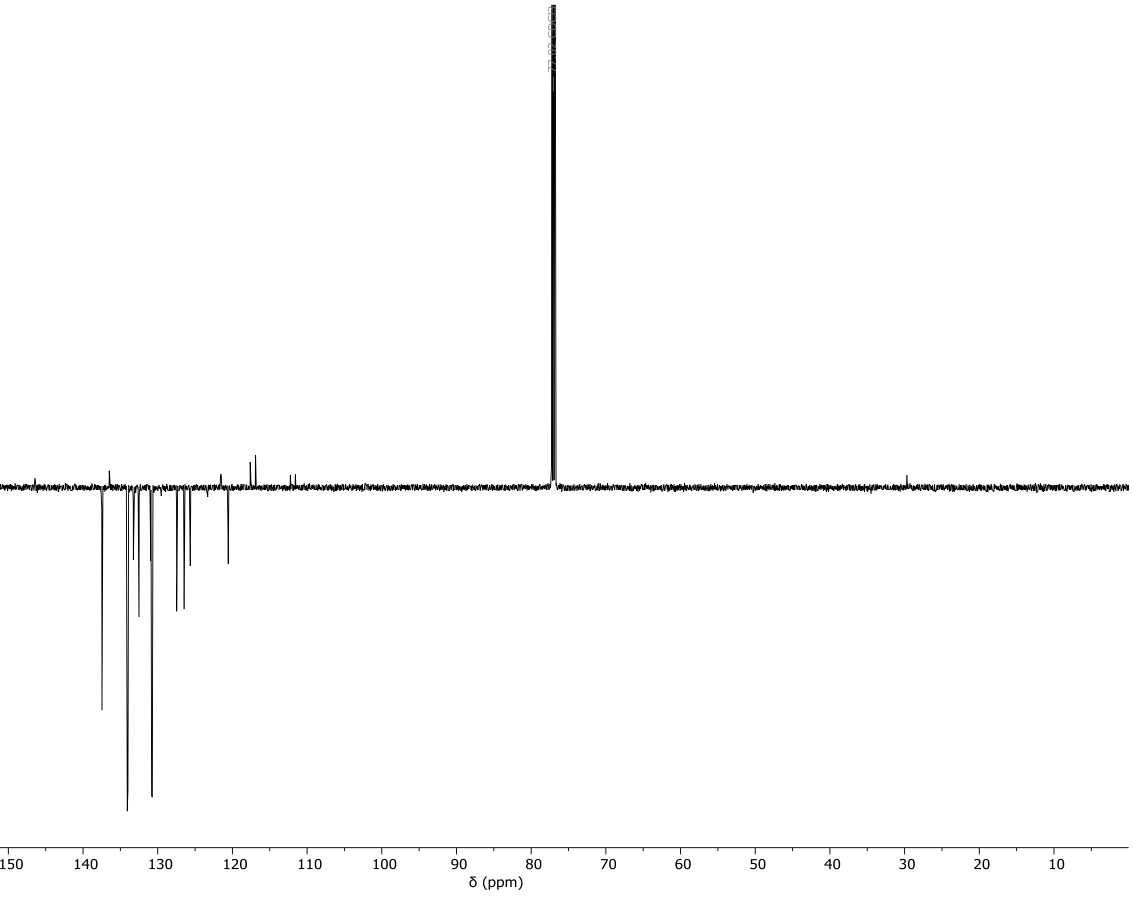

Figure S29. $^{13}C\{^{1}H\}$ DEPTQ NMR ($CDCl_3$, 151 MHz, 295 K) spectrum of **1′a**.

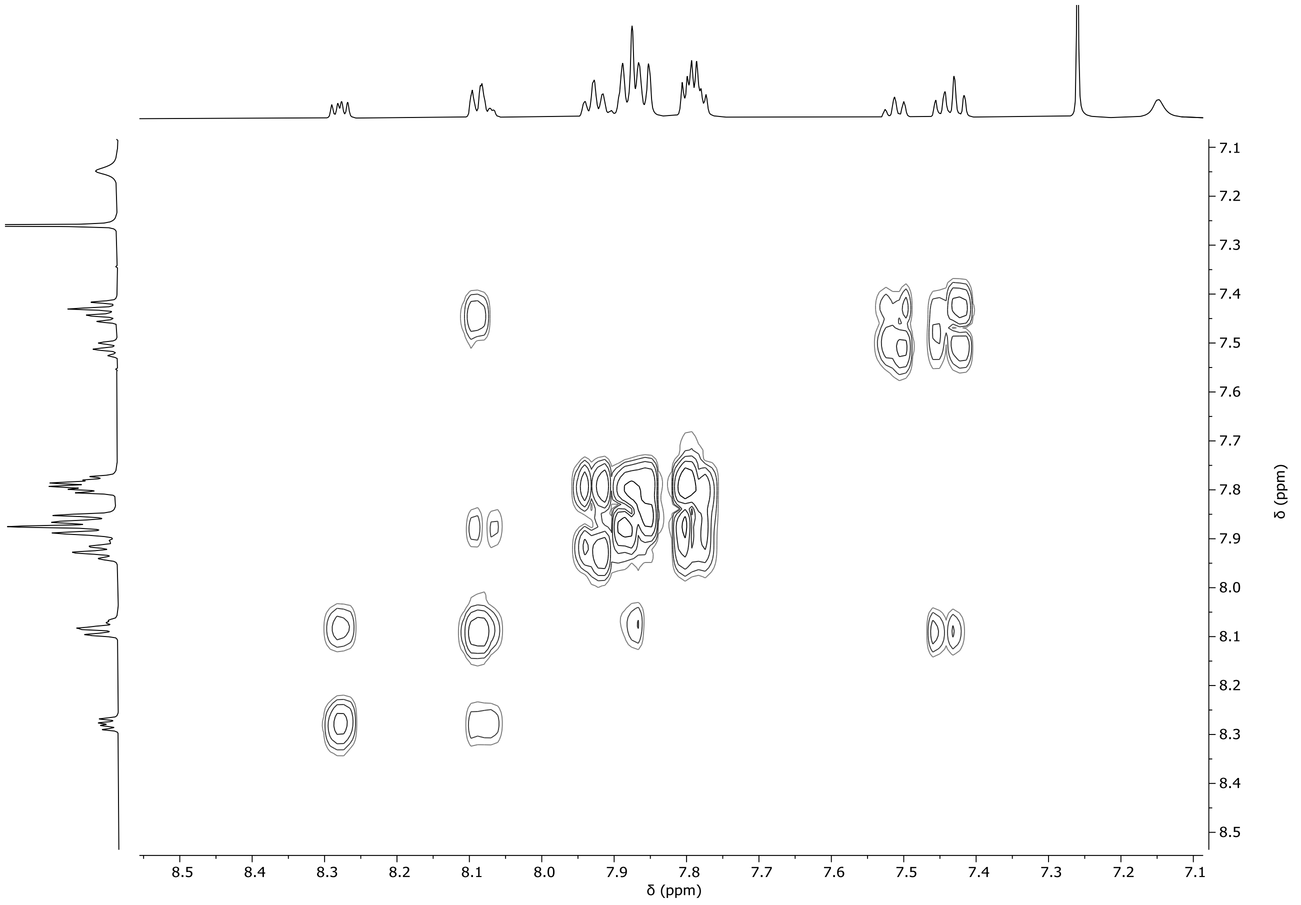

Figure S30. Partial COSY NMR ($CDCl_3$, 600 MHz, 295 K) spectrum of **1′a**.

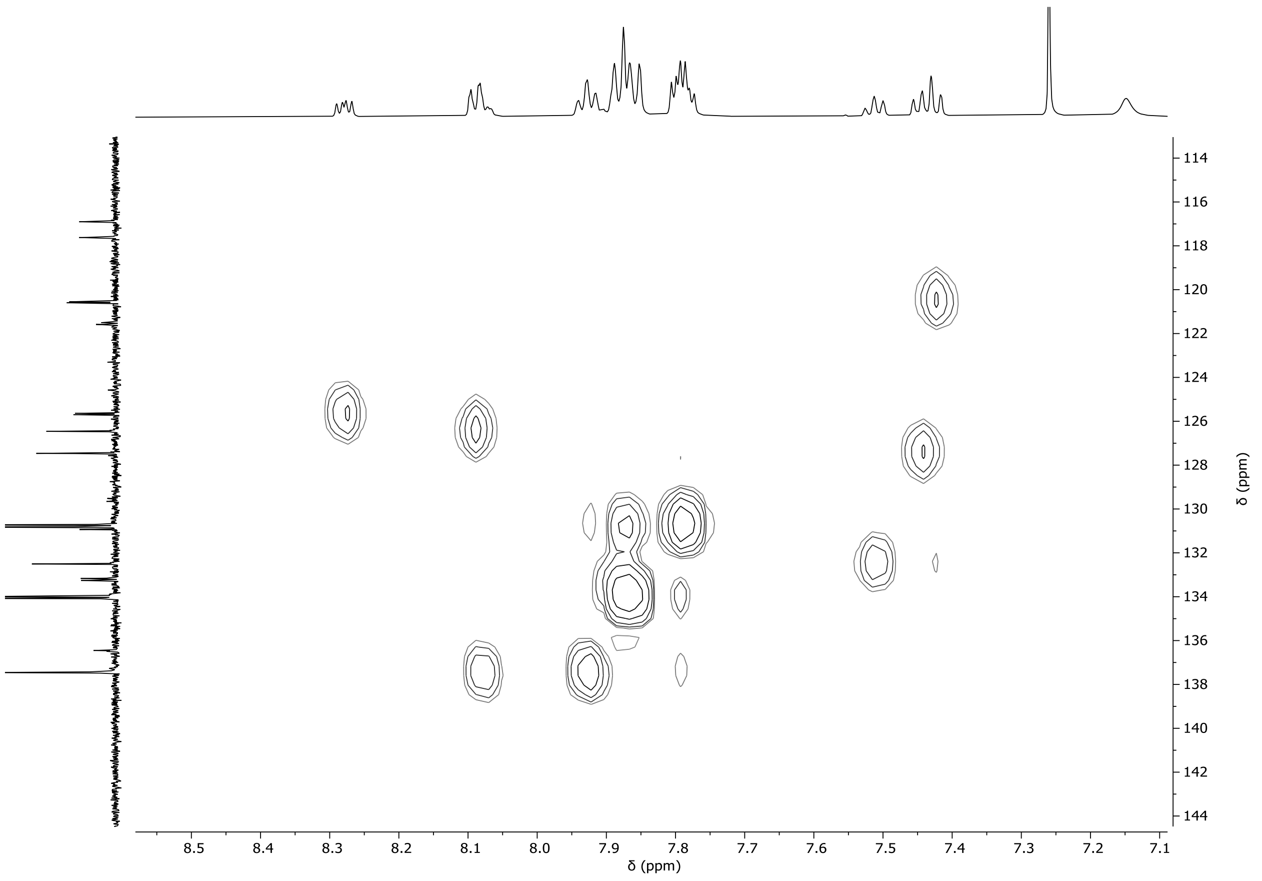

Figure S31. Partial ^1H-^{13}C HSQC NMR ($CDCl_3$, 600 MHz, 295 K) spectrum of **1′a**.

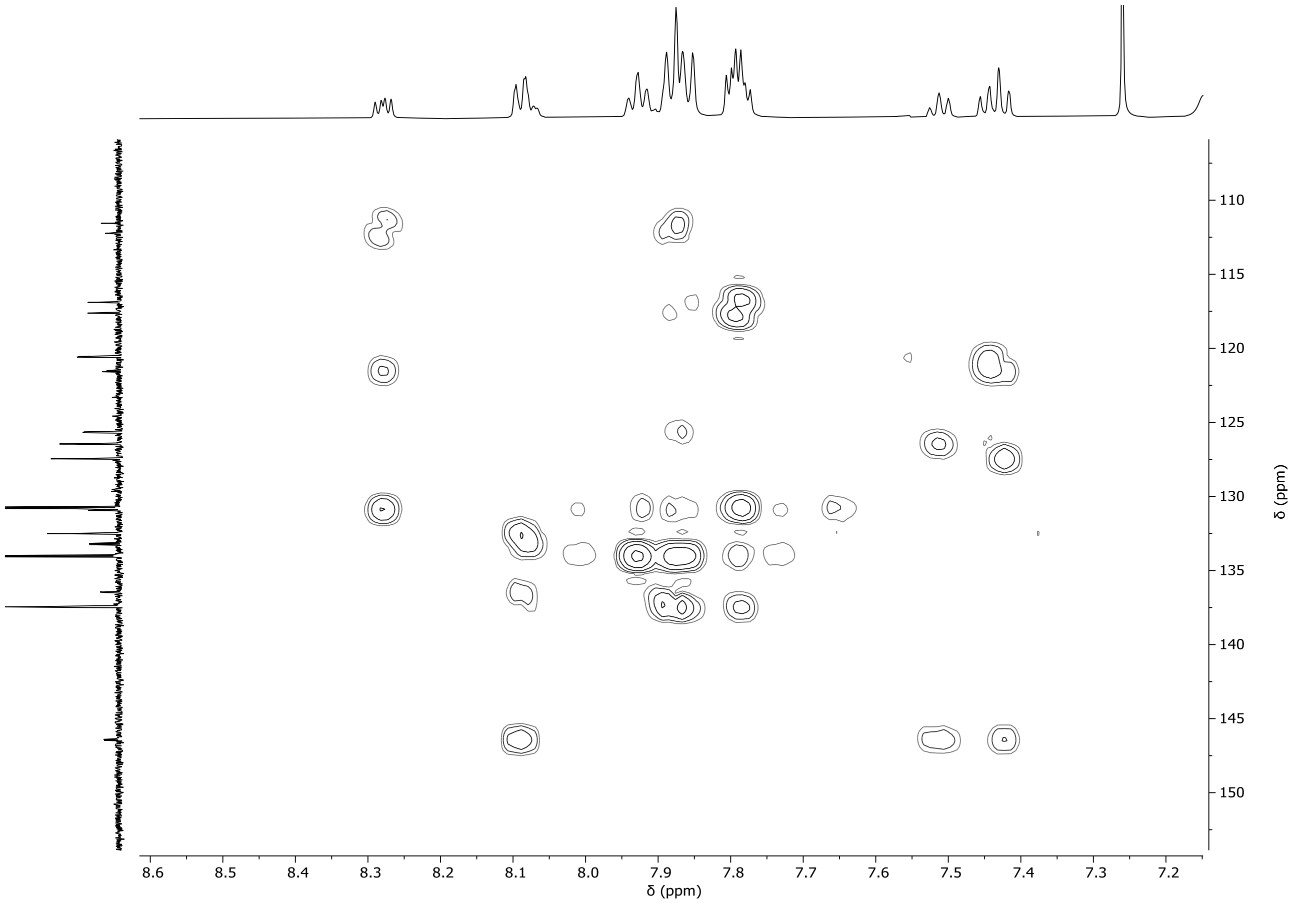

Figure S32. Partial ^{1}H-^{13}C HMBC NMR ($CDCl_3$, 600 MHz, 295 K) of **1′a**.

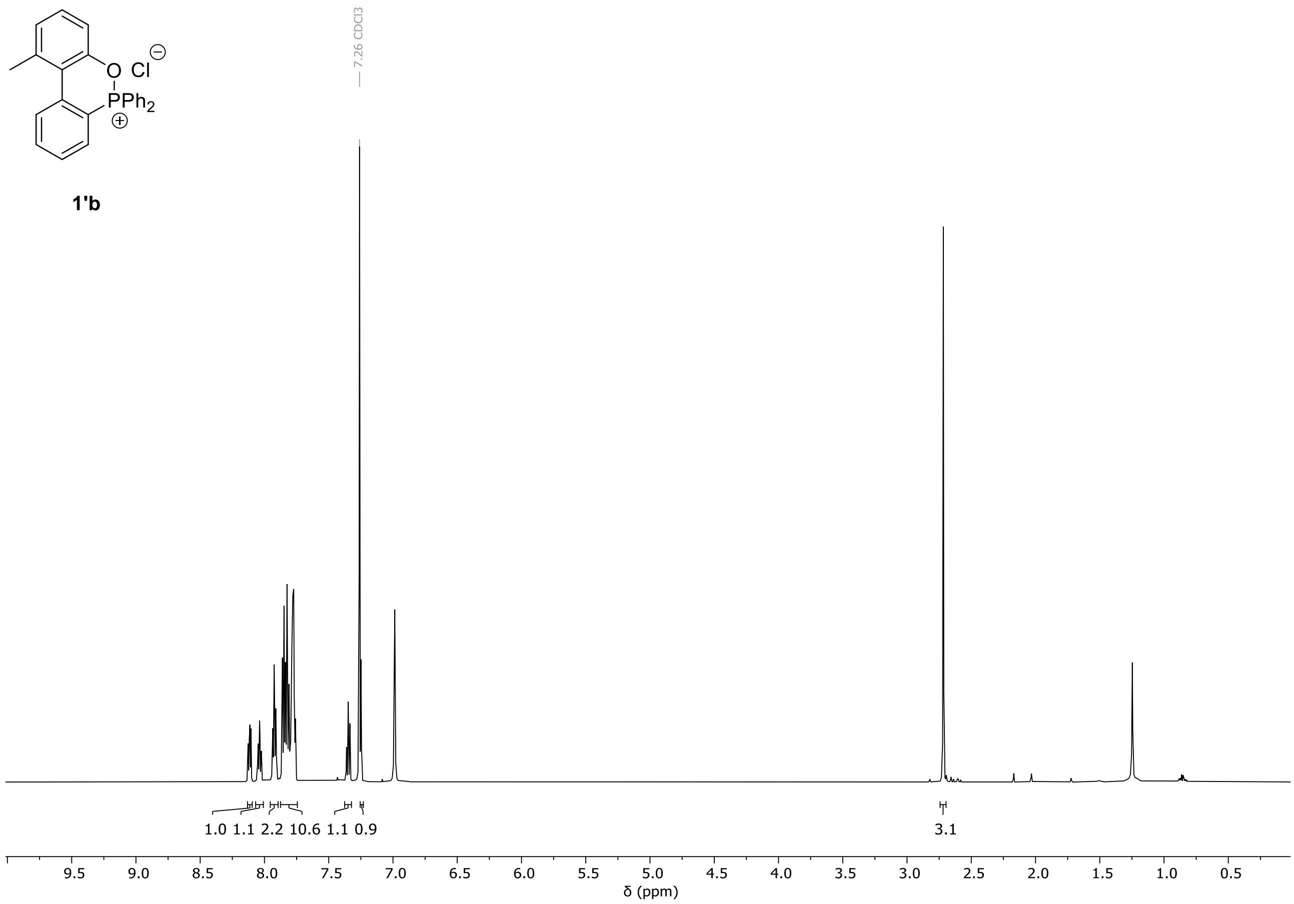

Figure S33. ^{1}H NMR ($CDCl_3$, 600 MHz, 295 K) spectrum of **1′b**.

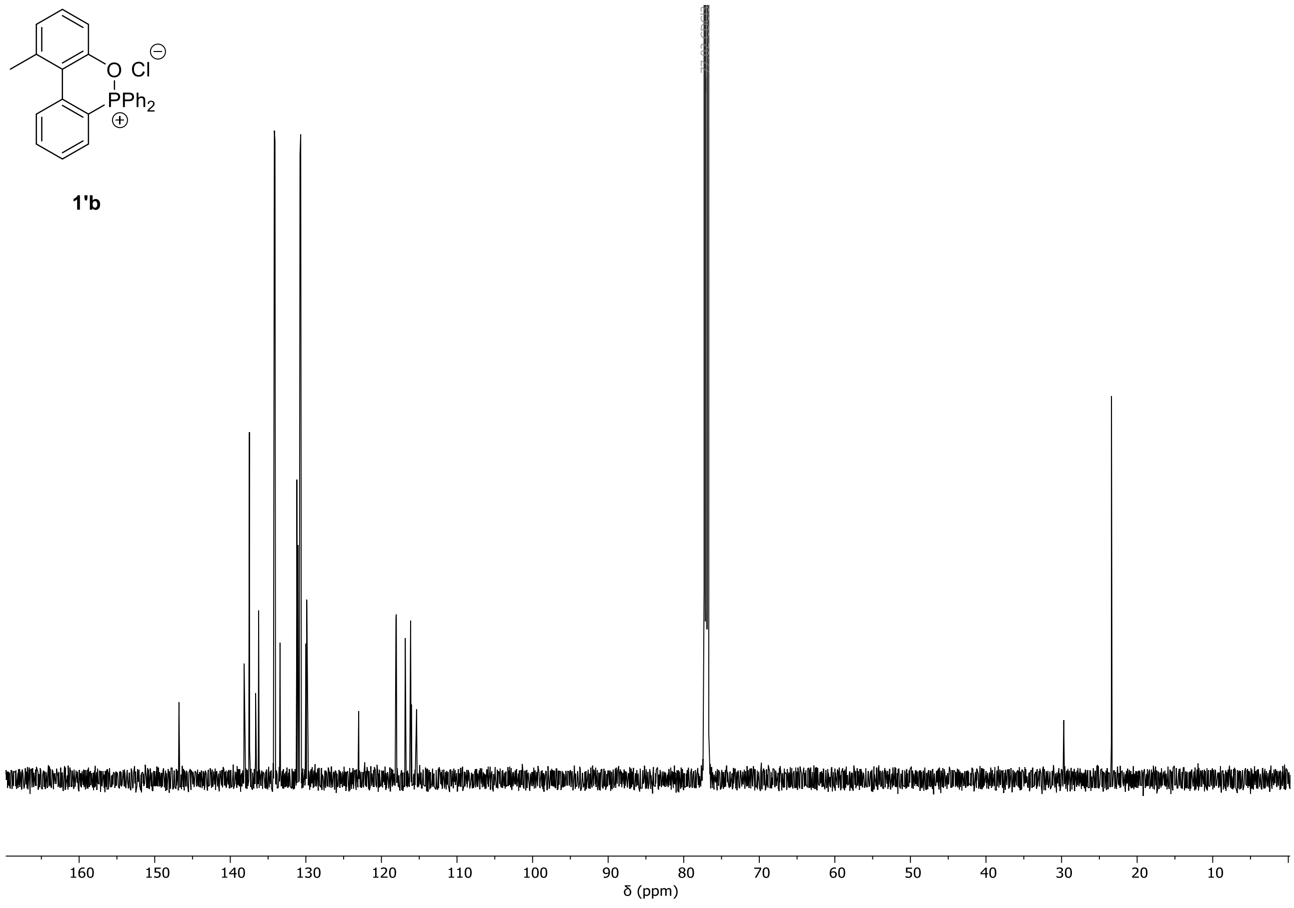

Figure S34. $^{13}C\{^{1}H\}$ NMR ($CDCl_3$, 151 MHz, 295 K) spectrum of **1′b**.

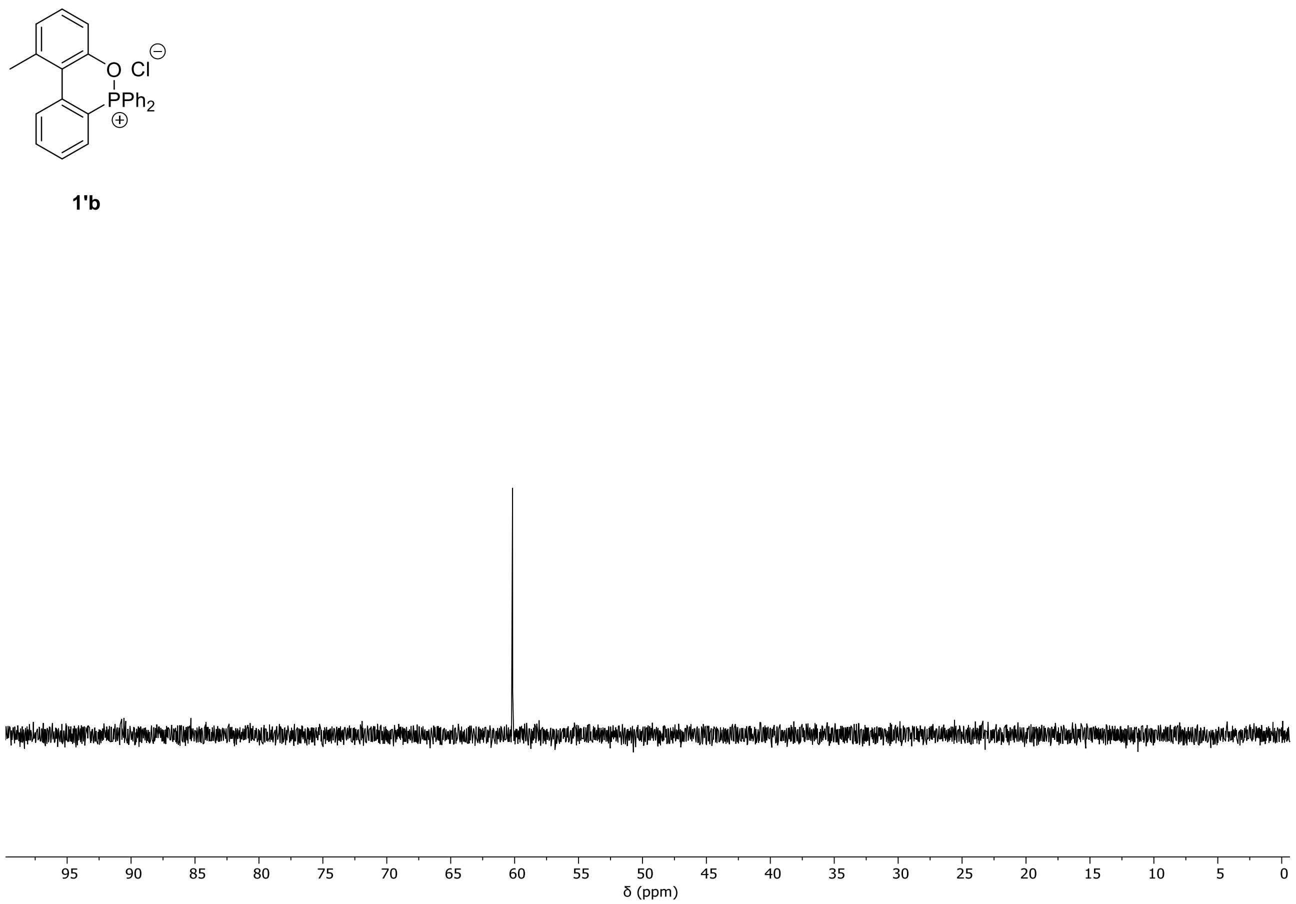

Figure S35. $^{31}P\{^{1}H\}$ NMR ($CDCl_3$, 151 MHz, 295 K) spectrum of **1′b**.

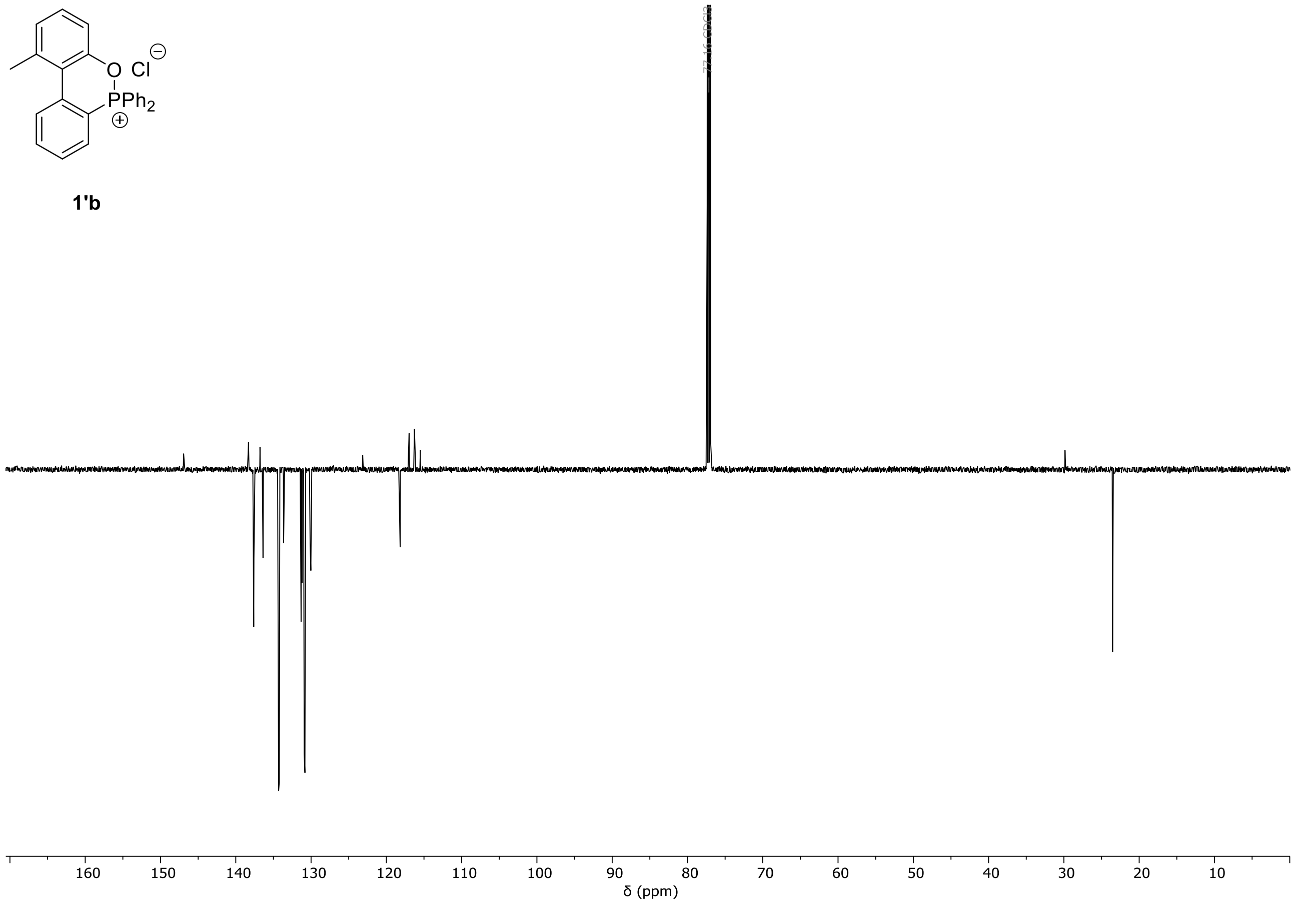

Figure S36. $^{13}C\{^{1}H\}$ DEPTQ NMR ($CDCl_3$, 151 MHz, 295 K) spectrum of **1′b**.

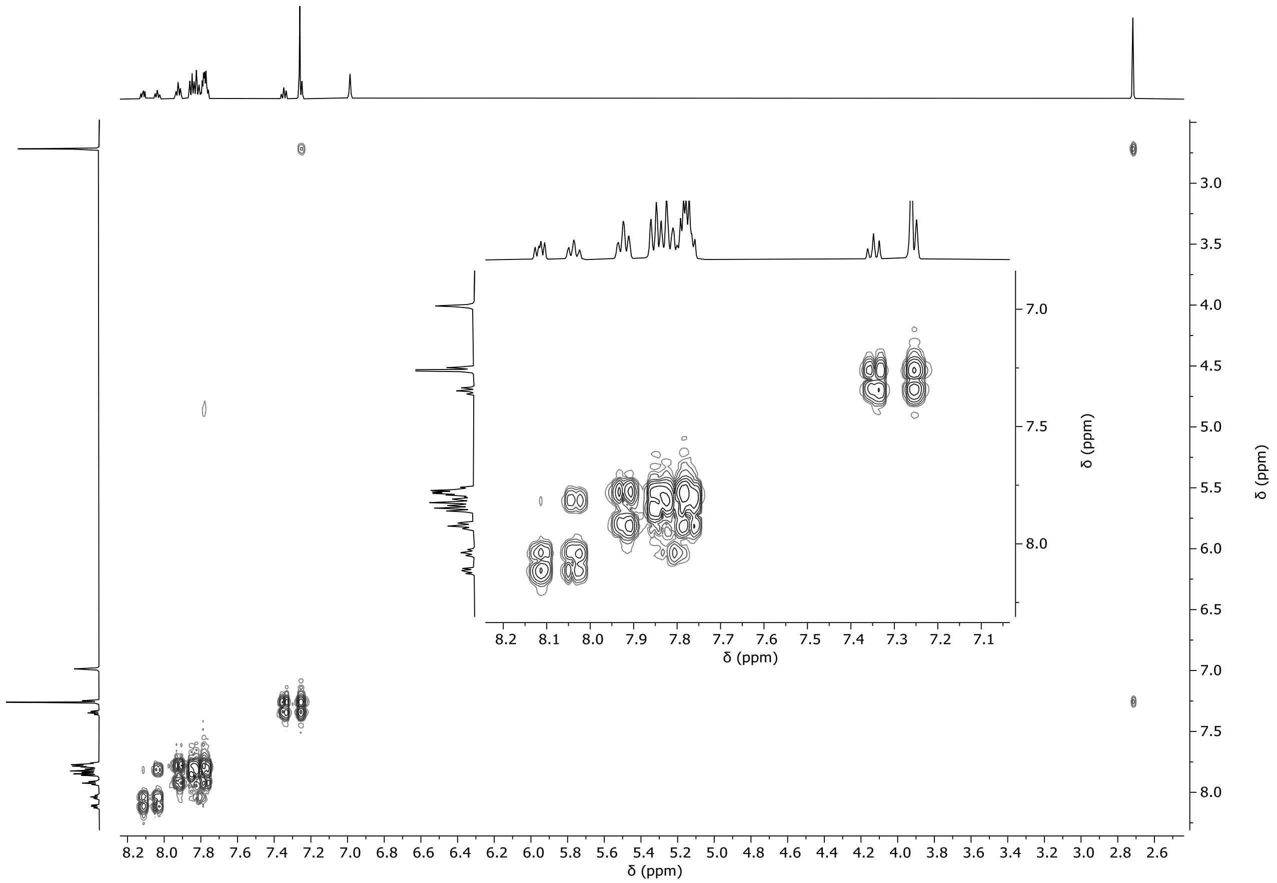

Figure S37. Partial COSY NMR ($CDCl_3$, 600 MHz, 295 K) spectrum of **1′b**.

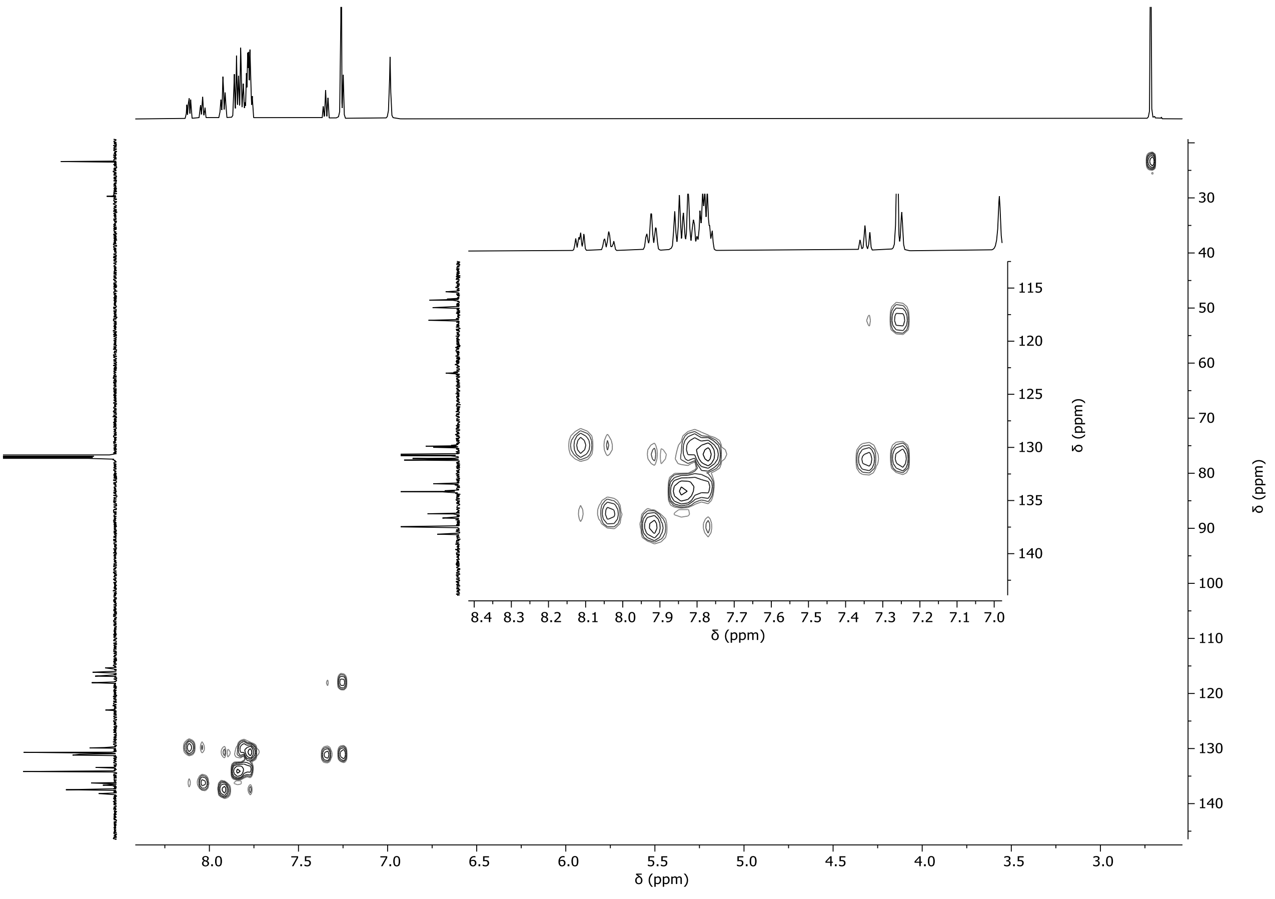

Figure S38. Partial ^{1}H-^{13}C HSQC NMR ($CDCl_3$, 600 MHz, 295 K) spectrum of **1′b**.

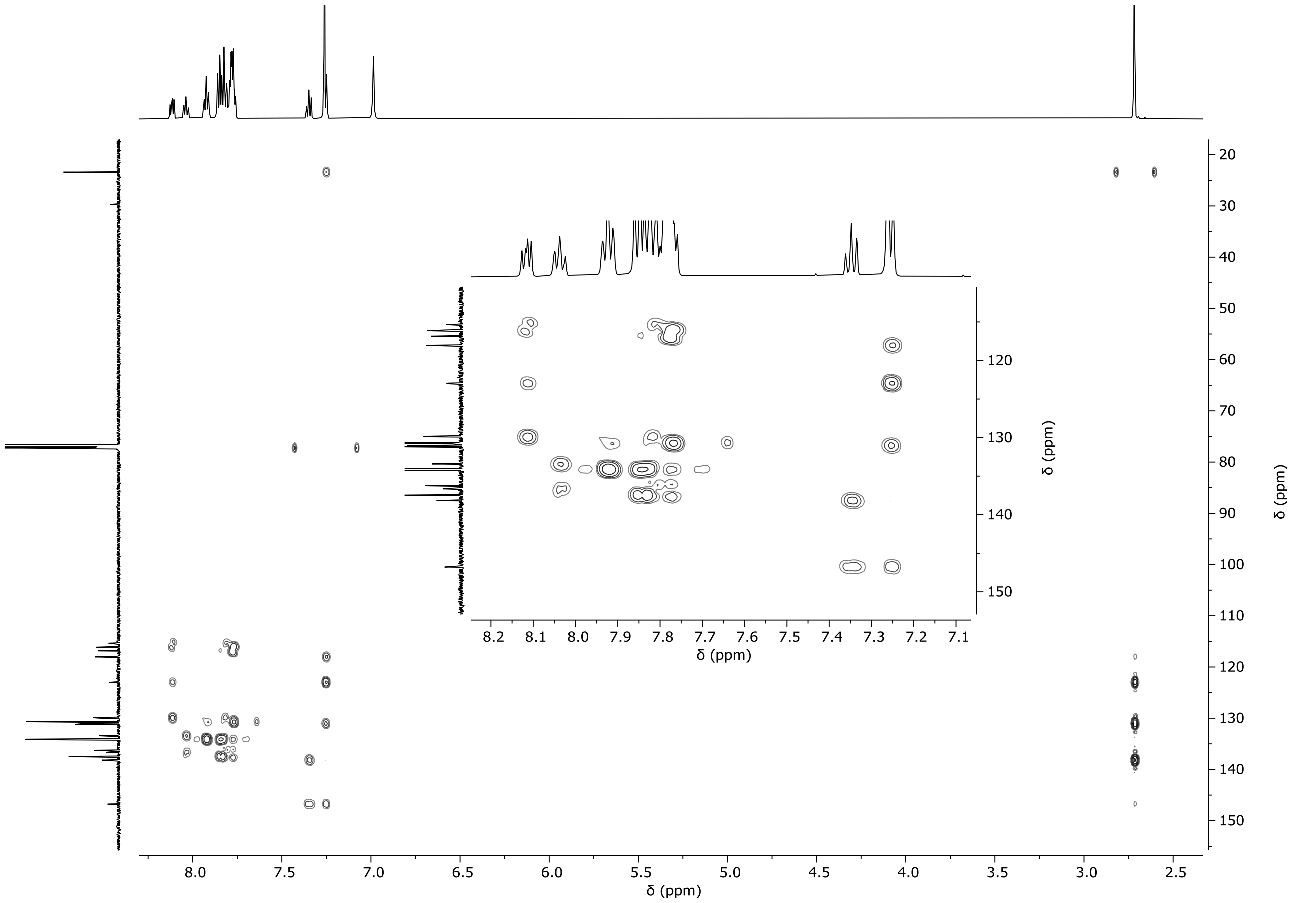

Figure S39. Partial ^{1}H-^{13}C HMBC NMR ($CDCl_3$, 600 MHz, 295 K) spectrum of **1′b**.

S8. References

S1. Zhang, H.-Y.; Yi, H.-M.; Wang, G.-W.; Yang, B.; Yang, S.-D.; Pd(II)-Catalyzed C(sp^2)–H hydroxylation with R_2(O)P-coordinating group. *Org. Lett.* **2013**, *15*, 6186–6189.

S2. Rigaku Oxford Diffraction, CrysAlisPro Software system, version 42, Rigaku Corporation, Oxford, UK, **2023**.

S3. a) Sheldrick, G. M. *Acta. Cryst.* **2015**, *A71*, 3–8; b) Sheldrick, G. M. *Acta. Cryst.* **2015**, *C71*, 3–8; c) Dolomanov, O. V.; Bourhis, L. J.; Gildea, R. J.; Howard, J. A. K.; Puschmann, H. *J. Appl. Cryst.* **2009**, *42*, 339–341.
